# Potential protective effects of *Phyllanthus emblica* L. extract on high-salt diet-induced hypertension: a combined analysis of gut microbiota and metabolomics

**DOI:** 10.3389/fphar.2026.1728643

**Published:** 2026-07-07

**Authors:** Mingzhu Peng, Zixin He, Jia Nie, Changhong Wei, Yamei Wu, Luming Qi, Jie Tang, Jun Chen

**Affiliations:** 1 Division of Head & Neck Tumor Multimodality Treatment, Cancer Center, West China Hospital, Sichuan University, Chengdu, China; 2 West China School of Nursing, Sichuan University, Chengdu, China; 3 Clinical Trial Center, West China Hospital, Sichuan University, Chengdu, China; 4 School of Health Preservation and Rehabilitation, and School of Ethnic Medicine, Chengdu University of Traditional Chinese Medicine, Chengdu, China; 5 Sichuan Academy of Chinese Medicine Sciences, Chengdu, China; 6 Department of Critical Care Medicine, Hospital of Chengdu University of Traditional Chinese Medicine, Chengdu, China

**Keywords:** gut metabolism, gut microbiota, high-salt diet, hypertension, Phyllanthus emblica

## Abstract

**Introduction:**

High-salt diet (HSD)-induced hypertension is a common form of hypertension and is closely associated with inflammation, target-organ injury, and gut microbiota dysbiosis. Natural products have shown potential in the prevention and treatment of hypertension, and regulation of the gut microbiota and its metabolites may represent an important therapeutic mechanism. Phyllanthus emblica L. (PE) is a medicinal plant with reported cardiovascular-protective and antihypertensive effects.

**Methods:**

In this study, a salt-sensitive rat model was used to systematically evaluate the effects of PE extract on blood pressure (BP), inflammatory responses, renal and vascular pathological changes, intestinal barrier function, gut microbiota composition, and metabolite profiles. The potential mechanisms of PE were further explored with a focus on the gut microbiota–metabolite axis.

**Results:**

PE intervention significantly alleviated the HSD-induced increase in BP, reduced the expression of the pro-inflammatory factors TNF-α and IL-1β, and improved renal and vascular tissue injury. PE also regulated the intestinal tight junction proteins Claudin-2 and ZO-1, suggesting an improvement in intestinal barrier function. Notably, high-dose PE extract restored HSD-induced gut microbiota dysbiosis, particularly by increasing the abundance of beneficial bacteria such as Lactobacillus. Metabolomic analysis showed that high-dose PE extract improved HSD-induced alterations in the intestinal metabolite profile, with eight bile acid metabolites being significantly reversed. Correlation analysis further suggested that the protective effects of PE may be associated with regulation of gut microbiota and their metabolites, especially through the bile acid pathway.

**Discussion:**

These findings suggest that PE extract may exert protective effects against HSD-induced hypertension by modulating the gut microbiota–metabolite axis, improving intestinal barrier function, reducing inflammation, and alleviating renal and vascular injury. This study provides preliminary experimental evidence for the potential application of PE in the prevention and treatment of salt-sensitive hypertension.

## 1 Introduction

Hypertension induced by high-salt diet (HSD) is a common and clinically significant disease type, characterized by an abnormally sensitive response of blood pressure (BP) levels to dietary salt intake (Chen L. et al., 2024). The pathogenesis and progression of this disease are closely associated with various factors such as genetic susceptibility, vascular dysfunction, and neuroendocrine regulatory disorders, significantly increasing the risk of cardiovascular and cerebrovascular events. Although multiple antihypertensive drugs are currently available for clinical treatment, existing therapeutic regimens still face limitations such as noticeable side effects and considerable variability in individual patient responses. This highlights the urgent need to explore safer and more effective alternative treatment options as a current research priority (Qi et al., 2024b).

In recent years, with in-depth exploration in the field of natural medicine research, the unique advantages of natural products and their active ingredients in multi-target regulation and holistic intervention have become increasingly prominent (Fan et al., 2025; Hu et al., 2025; He et al., 2025; Hou et al., 2024; Wang X. et al., 2025). Various natural medicinal extracts, as multi-component complex systems, have demonstrated broad prospects in the prevention and treatment of this type of hypertension due to their multiple pharmacological effects, including anti-inflammatory, antioxidant, vascular function improvement, and metabolic regulation. For example, Zeng et al. (Zeng et al., 2022) demonstrated that bitter melon extract effectively prevents HSD-induced hypertension by improving nitric oxide synthesis, regulating amino acid metabolism, and alleviating oxidative stress. These natural active components, through multi-pathway, multi-target synergistic mechanisms, provide important insights for the development of novel antihypertensive drugs and have become a research focus and development direction in the current field of hypertension prevention and treatment (Alblihy, 2024; Sadaqat et al., 2023).

*Phyllanthus emblica* L. (PE) is a botanical drug widely used in several traditional medical systems, including Tibetan and Uyghur medicine. In traditional medical practice, it has long been used to manage disease conditions associated with dizziness, abnormal blood circulation, and heat-related vascular syndromes, which may be related, to some extent, to the modern biomedical concept of hypertension (Prananda et al., 2023). Modern research has shown that PE is rich in various metabolites, including tannins, flavonoids, and vitamin C, endowing it with significant antioxidant, anti-inflammatory, and immunomodulatory effects (Prananda et al., 2023; Qi et al., 2020). Accumulating experimental evidence further suggests that PE may exert cardiovascular-protective effects through multiple mechanisms, including attenuation of oxidative stress and inflammation, improvement of vascular function, and regulation of the renin-angiotensin system (Yan et al., 2022; Zeng et al., 2023). Although several studies have reported beneficial effects of PE or its extracts in hypertension-related or cardiovascular-metabolic disorders, its specific effects and underlying mechanisms in high-salt diet-induced hypertension remain insufficiently understood.

A growing body of evidence indicates that gut microbiota dysbiosis plays an important role in hypertension by affecting microbial metabolite production, intestinal barrier integrity, and systemic inflammation (Mu et al., 2024; Naik et al., 2022; Wang and Hu, 2024; Zaher et al., 2024; Mansour et al., 2024). In our previous study, long-term HSD markedly reshaped gut microbiota composition, reduced beneficial bacteria, increased harmful bacteria, and promoted endotoxin translocation across the impaired intestinal barrier, thereby contributing to chronic inflammation, endothelial dysfunction, and elevated BP (Qi et al., 2024a). Restoring gut microbial and metabolic homeostasis may therefore represent a promising strategy for HSD-induced hypertension (Qi et al., 2024b). PE has also been reported by our group to modulate gut microbiota (Wang et al., 2024). However, whether and how the extract alleviates HSD-induced hypertension by reshaping the gut microbiota and its metabolite profile remains unknown.

Herein, using salt-sensitive rats (a specialized animal model for studying salt-sensitive hypertension), this study aims to elucidate the regulatory effects of PE extract on high-salt diet (HSD)-induced hypertension, employing an integrated approach of gut microbiome and gut metabolome analyses. Specifically, it will assess the extract’s impact on BP and renal pathology, explore its influence on gut microbiota composition, intestinal barrier function, and gut metabolites, and analyze the relationship between HSD-induced hypertension improvement and gut microbiota modulation. By investigating these mechanisms, the study seeks to provide novel insights and experimental evidence supporting the use of natural products, such as PE, to ameliorate HSD-induced hypertension.

## 2 Materials and methods

### 2.1 Materials and reagents

The dried fruits of PE were purchased from the Hehuachi Medicinal Material Market in Chengdu, China. These medicinal materials were identified by the professor Luming Qi from Chengdu University of Traditional Chinese Medicine as the dried ripe fruits of *P. emblica* L. The normal diet (0.3% NaCl) and high-salt diet (8.0% NaCl) were obtained from Keao Xieli Feed Co., Ltd. (Beijing, China). Standard chemical reference substances, including gallic acid (CHB231025), chebulagic acid (CHB240810), chebulic acid (CHB240332), geraniin (CHB240910), ascorbic acid (CHB231112) and ellagic acid (CHB240430), were purchased from Zorme Biotech Co., Ltd. (Chengdu, China), with all reagents having a purity >98%. Chromatography-grade methanol was purchased from Thermo Fisher Scientific (Shanghai) Co., Ltd. Deionized water for chromatographic analysis was produced using an ultrapure water system (Millipore, Merck Millipore, USA). Other analytical-grade reagents were provided by Chron Chemicals Co., Ltd. (Chengdu, China).

### 2.2 Preparation and chemical analysis of PE extract

The dried PE extracts were ground using a small herbal grinder and passed through an 80-mesh sieve to obtain a brown powder. An accurately weighed quantity of 100.0 g powder was placed in a conical flask and mixed with 50% aqueous ethanol (v/v) at a solid-to-liquid ratio of 1:20 (g/mL). The sealed flask was subjected to ultrasound treatment at a frequency of 40 kHz and a controlled temperature of 40 °C for 60 min. After processing, the mixture was centrifuged at 5,000 rpm for 15 min to collect the supernatant. The residue was re-extracted twice with a small volume of fresh solvent, and the supernatants from all three extractions were combined. The combined supernatant was concentrated under reduced pressure at 55 °C using a rotary evaporator, and finally dried to constant weight in a vacuum oven at 60 °C to obtain the solid extract, which was sealed and stored at −20 °C for subsequent use.

Two techniques were employed to characterize the chemical constituents of the medicinal materials. An ultra-high performance liquid chromatography quadrupole-orbitrap mass spectrometry (UPLC-QE-Orbitrap-MS) was used for untargeted profiling to comprehensively detect the chemical composition of the PE extract (Thermo Fisher Scientific, USA). Additionally, a TSQ plus triple quadrupole liquid chromatography-tandem mass spectrometry (UPLC/QqQ-MS) system was applied for targeted detection of specific known metabolitess identified within the extract (Thermo Fisher Scientific, USA). A certain amount of the obtained PE extract was dissolved in a 50% methanol solution and filtered through a 0.22 μm microporous membrane to prepare the test solution.

For targeted UPLC/QqQ-MS analysis, three independent extraction batches of PE extract were prepared and analyzed to assess the consistency of major representative metabolites across batches. The samples were chromatographically separated on an Accucore™ C_18_ chromatographic column (3.0 mm × 100 mm, 2.6 μm) and the column temperature was maintained at 30 °C. The mobile phase was composed of water containing 0.1% (v/v) formic acid (A) and acetonitrile (B), with an initial composition ratio of 95% A and 5% B. The following gradient program was adopted: 0–5 min, 5%–10% B; 5–20 min, 10%–25% B; 20–40 min, 25%–95% B; 40–45 min: 95%B. The flow rate of the mobile phase was 0.3 mL/min. The injection volume was 5 μL. For the UPLC/QqQ-MS analysis, the samples were chromatographically separated on an Acquity BEH C18 chromatographic column (3.0 mm × 100 mm, 2.6 μm) and the column temperature was maintained at 30 °C. The mobile phase was composed of water containing 0.1% (v/v) formic acid (A) and acetonitrile (B), with an initial composition ratio of 98% A and 2% B. The following gradient program was adopted: 0–2 min, 98%–90% A; 2–10 min, 90%–50% A; 10–15 min, 50%–5% A. The flow rate of the mobile phase was 0.3 mL/min. The injection volume was 2 μL.

### 2.3 Animal experiments and sample collection

Male Dahl salt-sensitive rats (6–8 weeks old, initial body weight approximately 180–200 g) were selected for this study because they develop a reliable hypertensive phenotype in response to high-salt intake. In addition, the use of male animals helped reduce potential variability associated with female sex hormone fluctuations during this initial mechanistic investigation (Fardoun et al., 2020). These rats were purchased from Vital River Laboratory Animal Technology Co., Ltd. (Beijing, China). After a 7-day acclimation period, the animals were randomly divided into four groups: Normal salt diet group (NSD, 0.3% NaCl, n = 6); HSD group (HSD, 8.0% NaCl, n = 6); low-dose PE group (PE_L, 200 mg/kg, n = 6); high-dose PE group (PE_H, 400 mg/kg, n = 6). The obtained PE extract was then re-dissolved in water for animal administration. The NSD and HSD groups received equal volumes of water by gavage. The low-dose and high-dose groups were administered 200 mg/kg and 400 mg/kg of PE extract via oral gavage, respectively. The selected doses were exploratory and based on preliminary experience and published study (Wang et al., 2024). All rats were allowed free access to food and water. Animals were housed under specific pathogen-free conditions, with a controlled temperature of 23 °C ± 1 °C, relative humidity of 50%–60%, and a 12-h light/dark cycle.

After 8 weeks of feeding, each rat was placed in an anesthesia chamber connected to a gas anesthesia machine (RWD Life Science Co., Ltd., Shenzhen, China). Anesthesia was induced with an initial isoflurane concentration of 3.5%. Once the animal showed no response to painful stimuli, the isoflurane concentration was maintained at 1.5%. A midline abdominal incision was made to expose the abdominal aorta. Blood was collected via the abdominal aorta using a blood collection tube while continuously administering isoflurane to maintain anesthesia. After blood collection, the isoflurane concentration was maintained until the animal’s respiration and heartbeat ceased. After terminal blood collection, aortic vessels, kidneys, colon, and fecal samples were collected for subsequent histopathological, intestinal barrier-related, microbiota, and metabolomics analyses. The isoflurane used in this experiment was purchased from RWD Life Science Co., Ltd. (Shenzhen, China). All experimental protocols were reviewed and approved by the Experimental Animal Ethics Committe of West China Hospital of Sichuan University (Approval No. 20240226107), adhering to international ethical standards and guidelines.

### 2.4 BP measurement

Tail-cuff plethysmography (CODA 4-channel system, Kent Scientific Corp., USA) was used as a non-invasive method to measure BP. Volume pressure recording was employed to measure BP based on tail blood volume. Rats were individually placed in small cages lined with sawdust, preheated to approximately 37 °C for 10 min, and then positioned in customized rodent restrainers with adjustable nosepieces and rear doors. The rats were placed in a prone position on a heating pad to maintain body temperature at 37 °C.

Blood pressure measurement was initiated after group allocation and the start of dietary/PE intervention, and was performed every other day throughout the experimental period until euthanasia. All measurements were conducted at approximately 9:00 a.m. in a quiet laboratory to minimize the influence of circadian variation and environmental stress. Systolic blood pressure (SBP) and diastolic blood pressure (DBP) were measured every other day for each group until euthanasia. Mean arterial pressure (MAP) was estimated using the formula: MAP = (SBP + 2 × DBP)/3. All measurements were performed by the same trained operator under standardized conditions to minimize variability. However, stress-related artifacts associated with the tail-cuff method could not be completely excluded.

### 2.5 Biochemical and histopathological analysis

Approximately 10 mL of blood was collected from the abdominal aorta of each rat at the end of the experiment and centrifuged at 3,000 r/min for 10 min at 4 °C to obtain plasma samples. The plasma was used for the measurement of inflammatory cytokines, including TNF-α, IL-6, IL-10, and IL-1β, using enzyme-linked immunosorbent assay kits provided by Elabscience Biotechnology Co., Ltd. (Wuhan, China).

After terminal blood collection, aortic vessels, kidney tissues, colon tissues, and fecal samples were further collected for subsequent analyses. The aortic vessels, kidneys, and colon tissues were fixed in 4% paraformaldehyde for 48 h and then transferred to 70% ethanol. The tissues were subsequently embedded in paraffin and sectioned into 5 μm slices. Hematoxylin and eosin (H&E) staining was performed on vascular, kidney, and colon sections for histological evaluation of tissue injury and morphological alterations.

### 2.6 Immunofluorescence and PCR analysis

Colon tissues were fixed in 4% paraformaldehyde for at least 24 h. The samples were then deparaffinized, rehydrated, subjected to antigen retrieval, and blocked for nonspecific binding. For immunofluorescence analysis of colon tissues, primary antibodies against ZO-1, occludin, claudin-1 and claudin-2 were diluted and incubated overnight at 4 °C. The sections were subsequently treated with FITC-labeled secondary antibodies and counterstained with DAPI. After staining, the sections were mounted with an anti-fade mounting medium and observed under a fluorescence microscope.

Total RNA was extracted from colon tissues, and complementary DNA (cDNA) was synthesized using a reverse transcription kit (Rongwei, Sichuan, China). The synthesized cDNA served as a template for quantitative real-time PCR (qPCR) to determine the relative expression levels of ZO-1, occludin, claudin-1 and claudin-2. The qPCR reactions were set up according to the manufacturer’s instructions under the following conditions: initial denaturation at 95 °C for 2 min, followed by 40 cycles of 95 °C for 15 s, 60 °C for 30 s, and 72 °C for 30 s β-actin or GAPDH was used as the reference gene, and the relative expression levels of target genes were calculated using the 2^(-ΔΔCt) method.

### 2.7 16S rRNA sequencing and analysis

Genomic DNA was extracted from fecal samples using a fecal DNA extraction kit (Cwbio, Jiangsu, China). The quality and concentration of the extracted DNA were assessed using agarose gel electrophoresis and a NanoDrop® ND-2000 spectrophotometer (Thermo Scientific Inc., USA). The V3-V4 region of the 16S rRNA gene was amplified using universal primers. The PCR products were purified, quantified, and sequenced on the Illumina NovaSeq 6,000 platform. Raw sequencing data were processed using the QIIME2 pipeline. Primer sequences and low-quality reads were removed according to the sequencing quality profiles. Denoising, quality filtering, and chimera removal were performed using DADA2 with the parameters set as trunc-len-f = 240, trunc-len-r = 220, trim-left-f = 0, and trim-left-r = 0. Amplicon sequence variants (ASVs) were generated after denoising. Taxonomic annotation was performed against the Silva database. For alpha and beta diversity analyses, all samples were rarefied to 20,000 reads per sample. Shannon and Chao1 indices were used to evaluate community richness and diversity, while beta diversity was calculated based on Bray–Curtis distance and visualized using principal coordinates analysis (PCoA) and non-metric multidimensional scaling (NMDS). These trimming parameters and rarefaction depth were selected according to sequencing quality and sequencing depth distribution to ensure the reliability of downstream diversity analyses. Differential abundance analysis was conducted using Linear Discriminant Analysis Effect Size (LEfSe) and volcano plots to identify significantly altered microbial taxa under experimental conditions or between groups.

### 2.8 Gut metabolomics detection and analysis

To analyze the gut metabolome, 200 mg of fecal samples were collected and metabolomics analysis was performed using UPLC-QE-Orbitrap-MS technique. Each sample was finely ground and subjected to ultrasonic extraction in an ice-water bath for 10 min. The resulting mixture was centrifuged at 13,000 rpm for 10 min, and 100 μL of the supernatant was collected. This was mixed with 200 μL of acetonitrile, vortexed for 60 s to precipitate proteins, and centrifuged again at 13,000 rpm for 10 min. The supernatant was transferred to a 1.5 mL EP tube and vacuum-dried. The dried residue was reconstituted in 200 μL of extraction solvent (Acetonitrile: Water = 4:1, v/v), vortexed for 60 s, and centrifuged at 13,000 rpm at 4 °C for 10 min. The final supernatant was filtered through a 0.22 μm membrane and prepared for metabolomics analysis. Gut metabolomics analysis was performed using fecal samples collected from individual rats, with each sample analyzed as an independent biological replicate (n = 6 per group). Quality control samples were included during the analytical run to monitor instrument stability and data consistency. PLS-DA was used as an exploratory multivariate approach for visualization of metabolic group separation.

### 2.9 Western blot analysis

Liver tissues were homogenized in RIPA lysis buffer containing protease inhibitor cocktail, and total protein was extracted according to the manufacturer’s instructions. Protein concentration was determined using a BCA protein assay kit. Equal amounts of protein (30 μg per lane) were separated by SDS–PAGE and then transferred onto PVDF membranes. After blocking with 5% non-fat milk for 1 h at room temperature, the membranes were incubated overnight at 4 °C with primary antibodies against FXR, CYP8B1, and GAPDH. After washing with TBST, the membranes were incubated with the corresponding HRP-conjugated secondary antibodies for 1 h at room temperature. Protein bands were visualized using an enhanced chemiluminescence detection system and quantified by densitometric analysis using ImageJ software. The protein expression levels of FXR and CYP8B1 were normalized to the internal control.

### 2.10 Data analysis

Numerical data are presented as mean ± standard deviation (SD). For animal experiments, each data point represents an individual biological replicate unless otherwise specified. Statistical analyses were performed using GraphPad Prism 9.0. Before one-way analysis of variance (ANOVA), normality and homogeneity of variance were assessed. After confirming that the relevant assumptions were met, one-way ANOVA followed by Tukey’s multiple-comparisons test was used for comparisons among groups. For correlation analysis, Spearman correlation was employed. A p value < 0.05 was considered statistically significant. For omics-based analyses, including the screening and presentation of differential metabolites and bacterial taxa, the present study mainly used exploratory statistical procedures. Differential features were screened using the criteria of P < 0.05 and |log2FC| > 1.2. Significant differences between the NSD group, HSD group, and intervention groups are indicated with asterisks: *P < 0.05, **P < 0.01, ***P < 0.001, ****P < 0.0001.

## 3 Results

### 3.1 Chemical profile analysis of PE extract

**FIGURE 1:**
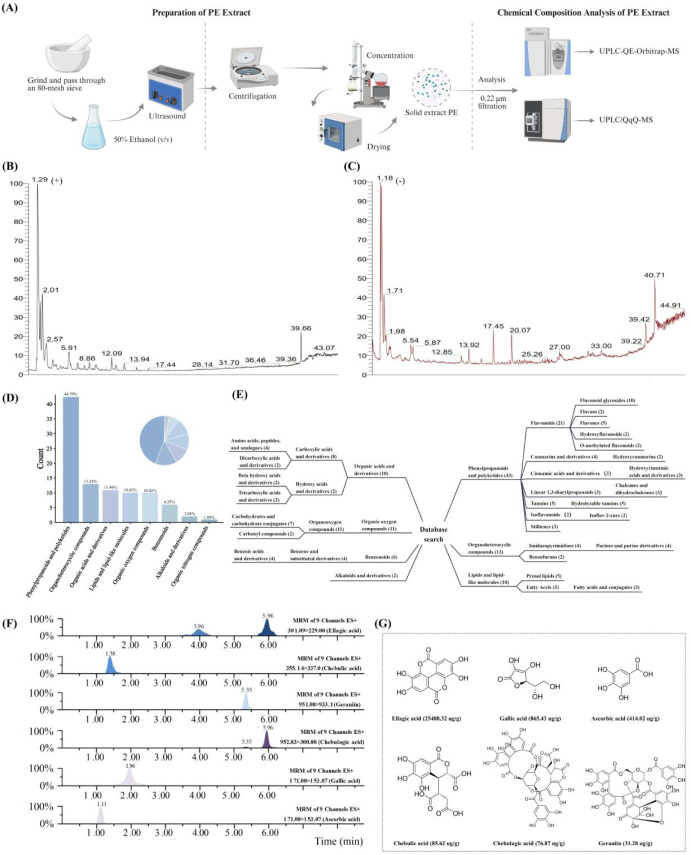
Effects of PE extract on hypertension and inflammatory levels. **(A)** procedure of animal experiments; **(B)** comparison analysis of body weight; **(C)** comparison analysis of MBP; **(D)** comparison analysis of SBP; **(E)** comparison analysis of DBP; **(F)** comparison analysis of TNF-α; **(G)** comparison analysis of IL-β; **(H)** comparison analysis of IL-10; **(I)** Comparison analysis of IL-6.

Following the preparation process, the extraction yield of the PE extract was determined to be 32% (w/w) (Figure 1A). As a complex mixture consisting of multiple metabolites, elucidation of the chemical profile of the PE extract is an essential precursor to the study of its antihypertensive activity in salt-sensitive rats. We first utilized UPLC-QE-Orbitrap-MS technology for the non-targeted analysis of chemical profile of the PE extract. The ion chromatograms obtained in positive and negative ion modes are presented in Figures 1B,C, respectively. By employing a screening process against the mzCloud database, followed by confirmation in the HMDB, 96 metabolites were successfully identified (Figure 1D; Supplementary Table S1). In PE extract, phenylpropanoids and polyketides constituted the most abundant category of metabolites, accounting for over 44% of the identified metabolites. This category primarily included flavonoid glycosides, flavans, chalcones and dihydrochalcones, and hydrolyzable tannins. Representative detected metabolites were ellagic acid, geraniin, chebulic acid and ascorbic acid. Other major categories encompassed amino acids, peptides, and analogues; benzoic acids and derivatives; and carbohydrates and carbohydrate conjugates. A dendrogram was generated to visualize the classification and relative distribution of the identified metabolites across major chemical categories, as shown in Figure 1E.

On the basis of non-targeted analysis, we performed a precise quantitative analysis of its representative constituents using UPLC/QqQ-MS in multiple reaction monitoring mode. Based on an extensive literature review, several metabolites with known pharmacological significance, such as ellagic acid, gallic acid, chebulagic acid, chebulic acid, geraniin and ascorbic acid were selected as the target analytes. Mass spectrometry detection parameters of components to be tested is shown in Table 1. Figure 1F depicts the specific multiple reaction monitoring chromatograms obtained for each of the candidate metabolites. Targeted quantification was performed on three independent extraction batches of PE extract, and the measured concentration ranges reflect the batch-to-batch variation of the major representative metabolites. Quantitative analysis revealed that ellagic acid (24.88–25.93 mg/g) was the most abundant phenolic metabolites in PE extract, followed by gallic acid (818.01–919.69 μg/g), ascorbic acid (404.23–425.14 μg/g), chebulic acid (75.61–94.86 μg/g), chebulagic acid (73.45–79.28 μg/g), and geraniin (21.68–40.28 μg/g) (Figure 1G). These results provided a preliminary chemical characterization of the PE extract and supported the identification of its major representative metabolites. However, more comprehensive fingerprint-based batch consistency evaluation will still be needed for future standardization and translational studies.

**TABLE 1:** Mass spectrometry detection parameters of components to be tested.

Metabolites	RT (min)	Ionization mode	Precursor ion (m/z)	Qualifier product ion (m/z)	Qualifier collision energy (V)	Quantifier product ion (m/z)	Quantifier collision energy (V)
Gallic acid	1.96	Positive	171	127.143	8.2	153.071	10.43
Ascorbic acid	1.11	Positive	177	95.125	11.57	141.125	6.85
Ellagic acid	5.96	Negative	301.088	227.012	31.83	229	25.64
Chebulic acid	1.38	Negative	355.138	205.071	18.77	337.071	10.86
Geraniin	5.35	Negative	951.075	301.143	45.82	933.113	20.33
Chebulagic acid	5.96	Negative	952.825	292.994	38.07	300.875	42.62

### 3.2 Effects of PE extract on Hypertension and Inflammatory Levels

**FIGURE 2:**
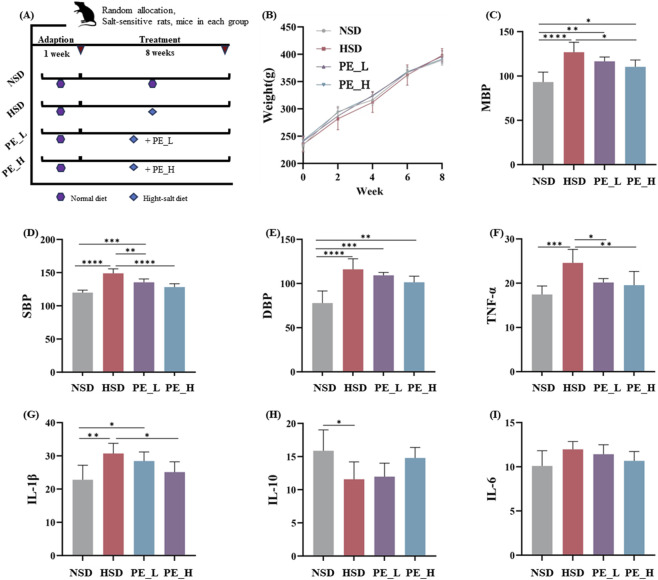
Effects of PE extract on hypertension and inflammatory levels. **(A)** Procedure of animal experiments; **(B)** Comparison analysis of body weight; **(C)** Comparison analysis of MBP; **(D)** Comparison analysis of SBP; **(E)** Comparison analysis of DBP; **(F)** Comparison analysis of TNF-α; **(G)** Comparison analysis of IL-β; **(H)** Comparison analysis of IL-10; **(G)** Comparison analysis of IL-6.

Building on the predictive insights derived from our previous network analysis, we specifically employed the salt-sensitive rat model for subsequent studies. This animal model uniquely recapitulates the salt-induced hypertensive phenotype observed in human salt-sensitive populations, and compared with conventional rodent models, it exhibits greater clinical relevance in investigating HSD-driven hypertension. Numerous studies have demonstrated that in this salt-sensitive rat model, BP typically increases significantly under prolonged exposure to HSD (Dube et al., 2023; Pravenec et al., 2024). To evaluate the therapeutic effect of PE on HSD-induced hypertension, we administered low-dose and high-dose PE interventions respectively over an 8-week HSD period, and monitored changes in BP as well as changes in related inflammatory factors (Figure 2A).

The results revealed that HSD significantly elevated MBP, SBP, and DBP compared to a normal diet (P < 0.001). Notably, PE extract interventions mitigated the rise in BP. The high-dose PE group exhibited the most pronounced antihypertensive effects, with significant reductions in MBP (P < 0.05) and SBP (P < 0.001) relative to the HSD group (Figures 2C–E). Importantly, body weight remained unaffected by either HSD or PE interventions throughout the study period (Figure 2B).

Given the established link between inflammation and hypertension (Patrick et al., 2021), we further assessed the levels of inflammatory markers, including TNF-α, IL-1β, IL-6, and IL-10 (Figures 2F–I). HSD significantly elevated TNF-α (P < 0.001) and IL-1β (P < 0.01), while reducing IL-10 levels (P < 0.05), indicating heightened systemic inflammation. PE extract interventions alleviated these inflammatory effects. Low-dose PE significantly reduced TNF-α levels (P < 0.05), whereas high-dose PE further decreased TNF-α (P < 0.01) and IL-1β (P < 0.05), while significantly increasing IL-10 levels. Interestingly, IL-6 levels were unaffected by either HSD or PE interventions. Collectively, these findings support an association between PE intervention and partial improvement in BP and selected inflammatory parameters under the present experimental conditions.

### 3.3 Effects of PE Extract on Pathological Features of Kidneys and Blood Vessels

Hypertension induced by a HSD fundamentally involves pathological alterations in the kidneys and blood vessels, which are pivotal mechanisms contributing to its progression. To investigate these effects, this study utilized HE staining to assess structural changes in renal and vascular tissues of salt-sensitive rats after 8 weeks of HSD exposure and interventions with different doses of PE extract.

**FIGURE 3:**
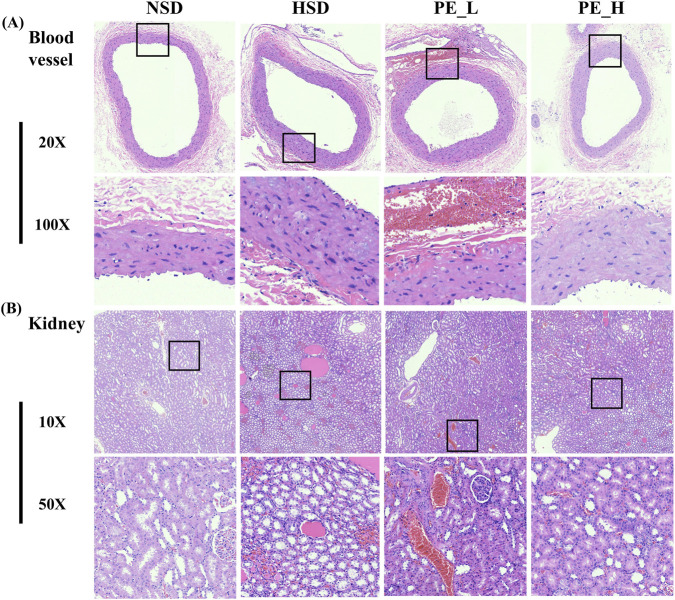
Effects of PE Extract on Pathological Features of Kidneys and Blood Vessels. **(A)** HE staining of Blood Vessels; **(B)** HE staining of Kidneys.

In the NSD group, the vascular wall structure was well-preserved, with cells in the media and adventitia arranged in an orderly fashion, and no signs of thickening or inflammation. In contrast, the HSD group exhibited severe vascular irregularities, including disorganized cellular arrangements in the media, extensive inflammatory cell infiltration, endothelial dysfunction, and prominent thickening of the adventitia. Collagen deposition and smooth muscle proliferation in the media produced an “onion-skin” pattern characteristic of advanced vascular pathology. PE extract intervention ameliorated these alterations, with both low- and high-dose groups showing reduced inflammatory infiltration and improved vascular structure. Notably, the high-dose group demonstrated nearly complete restoration of endothelial and media cell organization, and the adventitia structure resembled that of the NSD group (Figure 3A).

For kidney tissue, the NSD group maintained intact glomerular architecture, organized renal tubules, and no detectable interstitial inflammation or lumen casts. However, HSD caused substantial renal damage, including disorganized tubules, enlarged lumens, interstitial inflammation, fibrosis, and edematous proximal convoluted tubules and collecting ducts. These changes were accompanied by loose cytoplasm and altered tubule casts, indicative of impaired renal function. PE extract treatment substantially alleviated these pathological changes, with both dosing groups showing diminished fibrosis and inflammation. Strikingly, high-dose PE intervention nearly normalized glomerular and tubular structures, drastically reducing inflammatory reactions and restoring the organization of renal tubules and glomeruli (Figure 3B).

These findings demonstrate that HSD profoundly disrupts the structural integrity of blood vessels and kidney tissues, culminating in endothelial dysfunction and renal impairment. PE extract, particularly in high doses, exhibits significant protective effects by mitigating vascular and renal damage, underscoring its therapeutic potential against HSD-induced hypertension.

### 3.4 Effects of PE Extract on Intestinal Barrier Function

**FIGURE 4:**
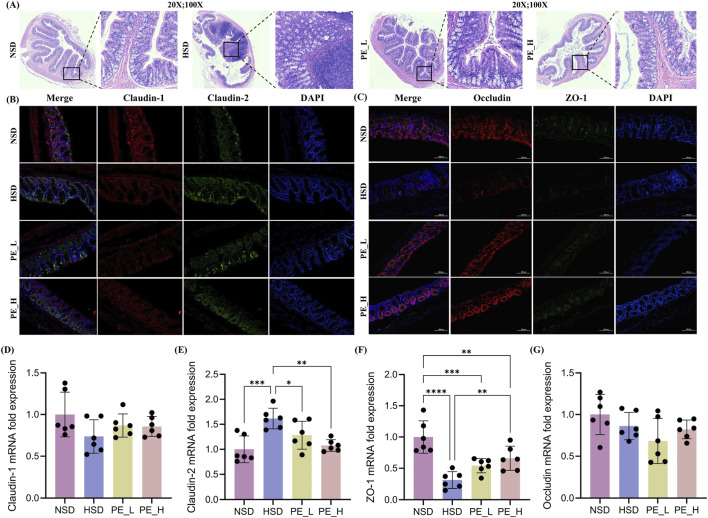
Effects of PE Extract on Intestinal Barrier Function. **(A)** HE staining of colon tissue sections; **(B)** Changes in Claudin-1 and Claudin-2 immunofluorescence; **(C)** Changes in Occludin and ZO-1 immunofluorescence; **(D)** Relative expression of Claudin-1 gene; **(E)** Relative expression of Claudin-2 gene; **(F)** Relative expression of ZO-1 gene; **(G)** Relative expression of Occludin gene.

The integrity of the intestinal barrier is a critical factor in mitigating hypertension induced by an HSD (Martel et al., 2022). By preventing the systemic entry of endotoxins, pro-inflammatory mediators, and other metabolically disruptive molecules, the intestinal barrier preserves overall metabolic homeostasis. To elucidate the effects of HSD and PE extract on intestinal function, an 8-week intervention was conducted in salt-sensitive rats. Histological evaluation using HE staining revealed an intact mucosal architecture and well-defined villi in rats fed an NSD. In contrast, HSD exposure caused substantial intestinal damage, including shortened and detached villi, disrupted enterocyte structures, and compromised barrier function. Both PE_L and PE_H interventions effectively alleviated these abnormalities, with the high-dose group demonstrating near-complete restoration of villi morphology to a state comparable to that of the NSD group (Figure 4A). Critical components of intestinal tight junctions, such as ZO-1 and Claudin-2, were further analyzed to assess barrier integrity. HSD markedly downregulated ZO-1 protein levels while simultaneously upregulating Claudin-2 expression, indicative of increased permeability and barrier dysfunction. Immunofluorescence staining suggested that PE_L and PE_H partially modulated the expression patterns of these proteins, with the clearest changes observed for ZO-1 and Claudin-2. However, Claudin-2 and Occludin protein levels showed no significant changes between the HSD and PE treatment groups (Figures 4B,C). Consistent with these findings, qPCR analysis of relative mRNA expression levels corroborated the trends observed at the protein level (Figures 4D–G). In summary, the histological and molecular results suggest that PE extract partially ameliorated HSD-induced intestinal injury and altered several barrier-associated markers. These findings support a potential association between intestinal barrier-related changes and the protective effects of PE under the present experimental conditions.

### 3.5 Effects of PE Extract on Gut Microbiota Structure and Composition

The gut microbiota plays a pivotal role in maintaining intestinal homeostasis and modulating systemic processes, including the development and progression of hypertension (Li et al., 2020). Disruption of microbial balance, often caused by environmental or dietary factors, can exacerbate inflammation and metabolic dysfunction, contributing to hypertension. To evaluate whether PE extract modulates gut microbiota in hypertension induced by a HSD, this study employed high-throughput 16S rDNA sequencing to assess microbial diversity, composition, and community structure in the NSD, HSD, and high-dose PE intervention groups.

**FIGURE 5:**
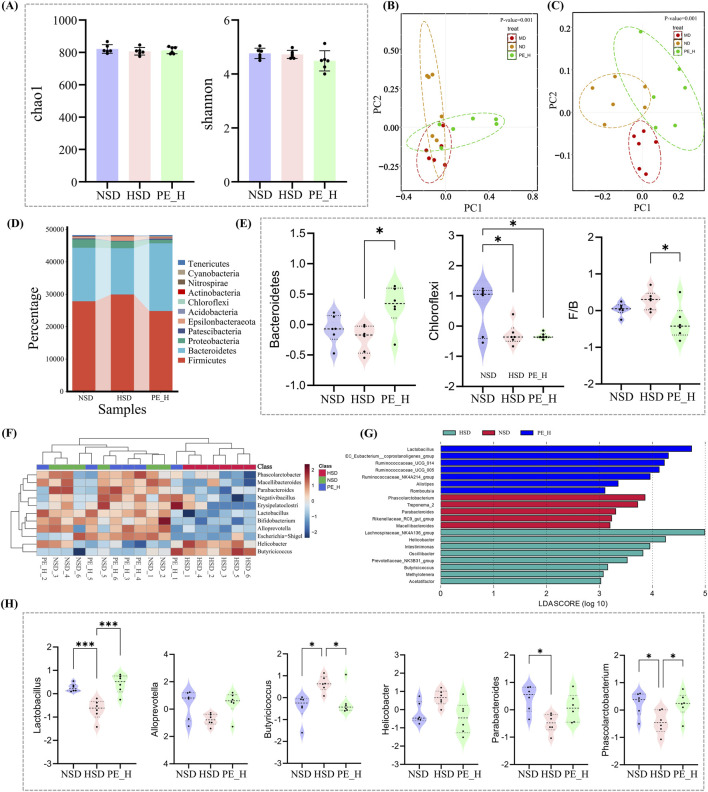
Effects of PE Extract on Gut Microbiota Structure and Composition. **(A)** Comparison of gut microbiota diversity; **(B)** PCoA analysis of different groups; **(C)** NMDS analysis of different groups; **(D)** Changes in phylum-level gut bacterial proportions; **(E)** Phylum-level changes in gut bacterial abundance; **(F)** Cluster heatmap analysis; **(G)** Lefse analysis; **(H)** Comparison analysis of key bacterial genera.

Sequencing depth was generally adequate and consistent across samples, with raw reads ranging from 79,461 to 80,215 per sample and effective reads ranging from 74,515 to 77,533 per sample (Supplementary Table S2). The α-diversity, a measure of microbial richness and evenness, was evaluated using chao 1 and shannon indices. The results indicated that neither HSD nor PE extract intervention significantly altered α-diversity, suggesting that the overall microbial richness remained unaffected (Figure 5A). In contrast, β-diversity, which reflects differences in microbial community composition, showed significant variation among the groups. Using Bray-Curtis distance metrics, PCoA, and NMDS plots, we observed substantial clustering separation in the HSD group, indicating that HSD profoundly altered the gut microbiota structure (P = 0.001). Notably, high-dose PE extract intervention partially restored the microbiota composition and structure, aligning it closely with the NSD group, thereby demonstrating its therapeutic potential in reversing HSD-induced dysbiosis (Figures 5B,C).

Taxonomic analysis at the phylum level revealed that Bacteroidetes and Firmicutes were the dominant bacterial phyla across all groups (Figure 5D). HSD significantly reduced the abundance of Bacteroidetes while increasing the Firmicutes-to-Bacteroidetes (F/B) ratio, a well-established marker of gut microbiota imbalance. The high-dose PE extract intervention reversed these shifts, significantly restoring Bacteroidetes levels and reducing the F/B ratio, which may indicate improved microbial homeostasis. Furthermore, HSD also reduced the abundance of less dominant phyla such as Chloroflexi, a trend that was partially mitigated by PE extract treatment (Figure 5E). These findings suggest that HSD profoundly disrupts the microbial community at the phylum level, while high-dose PE extract intervention plays a key role in partially remodelling gut microbiota composition.

**FIGURE 6:**
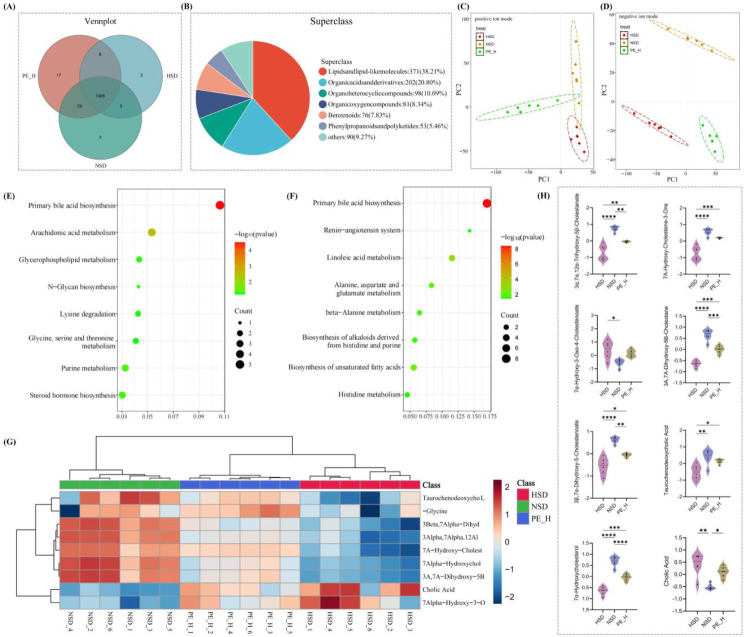
Effects of PE Extract on Gut Metabolism. **(A)** The vebbplot of metabolites in different groups; **(B)** Classification of identified metabolites; **(C)** PLS-DA analysis in positive ion mode; **(D)** PLS-DA analysis in negative ion mode; **(E)** KEGG analysis of differential metabolites between NSD and HSD groups; **(F)** KEGG analysis of differential metabolites between HSD and PE_H groups; **(G)** Cluster heatmap analysis; **(H)** Comparison analysis of important differential metabolites.

At the genus level, differential abundance analysis, heatmap clustering, and LEfSe revealed more granular insights into microbial changes. HSD significantly reduced the abundance of 22 genera and increased 9 genera (P < 0.05), emphasizing its disruptive effect. In contrast, high-dose PE extract intervention upregulated the abundance of 18 genera and downregulated 15 genera (P < 0.05) (Supplementary Table S3). Heatmap analysis further underscored these findings, where HSD samples formed distinct clusters indicative of dysbiosis, while samples from the NSD and high-dose PE groups clustered together, reflecting restored microbial composition (Figure 5F). LEfSe analysis highlighted the enrichment of harmful bacteria such as Lachnospiraceae_NK4A136_group and *Helicobacter* in the HSD group, whereas high-dose PE intervention increased beneficial genera, including *Lactobacillus*, renowned for its role in gut health. Visualization of key genera provided additional insight into potential candidate targets for future therapeutic applications (Figures 6G,H).

These findings collectively demonstrate that HSD profoundly alters gut microbiota structure, reducing beneficial bacteria while promoting harmful taxa associated with intestinal and systemic dysfunction. High-dose PE extract intervention significantly ameliorates these disturbances, re-establishing gut microbial homeostasis and suggesting a potential association between microbiota modulation and improved hypertensive phenotypes.

### 3.6 Effects of PE Extract on Gut Metabolism

Gut metabolites, the products of microbial metabolism, are critical mediators of host physiology and can influence BP regulation through diverse biochemical pathways (Duttaroy, 2021). In this study, we utilized UPLC-QE-Orbitrap-MS to examine the impact of an HSD and high-dose PE extract intervention on gut metabolites in salt-sensitive rats. After data cleaning and preprocessing, metabolites with a relative standard deviation (RSD) of less than 30% were retained for subsequent analyses, ensuring robust and reliable results.

A Venn diagram of the identified metabolites revealed that 1,405 metabolites were shared among the NSD, HSD, and PE intervention groups, while 17 metabolites were unique to the high-dose PE intervention group (Figure 6A). These metabolites were categorized into six principal groups: lipids and lipid-like molecules (38.21%), organic acids and derivatives (20.80%), organic heterocyclic metabolites (10.09%), benzene and substituted derivatives (7.83%), and organic oxygen metabolites (8.34%) (Figure 6B). This classification underscores the heterogeneity of gut-derived metabolites, reflecting their multifaceted roles in energy, lipid, and amino acid metabolism.

To evaluate global metabolic changes among the experimental groups, partial least squares discriminant analysis (PLS-DA) was performed. The results demonstrated significant separation of metabolic profiles in the NSD, HSD, and high-dose PE groups under both positive and negative ion modes, indicating that HSD treatment induced profound metabolic disruptions, which were markedly ameliorated by high-dose PE extract intervention (Figures 6C,D).

Subsequent metabolomic profiling based on the KEGG database identified 452 metabolites across all groups. Eight weeks of HSD treatment significantly downregulated 117 metabolites and upregulated 82 (P < 0.05). KEGG pathway analysis highlighted that these alterations were predominantly concentrated in the bile acid metabolism pathway (Figure 6E). High-dose PE extract intervention reversed these metabolic disruptions, with 73 metabolites downregulated and 181 significantly upregulated (P < 0.05) (Supplementary Table S4). Notably, the metabolic changes following PE intervention were also prominently associated with bile acid metabolism, highlighting this pathway as one of the major metabolic features identified under the present dataset. (Figure 6F).

Further analysis of bile acid metabolites clearly distinguished sample groups based on differential metabolite abundance (Figure 6H). Visualization of key bile acid metabolites provided additional insights, laying the groundwork for future investigations into their functional roles (Figure 6G). Among the bile acid-related metabolites, cholic acid was increased in the HSD group and was reduced after PE intervention, whereas several other bile acid-related metabolites, including 7α-hydroxycholesterol, 3α,7α-dihydroxy-5β-cholestane, 7α-hydroxy-3-oxo-4-cholestenoate, 3α,7α,12α-trihydroxy-5β-cholestanate, and 3β,7α-dihydroxy-5-cholestenoate, showed an opposite trend and were partially restored by PE treatment. These findings suggest that HSD disrupted bile acid-related metabolic balance, whereas PE intervention partially reversed these alterations.

In summary, high-dose PE extract significantly mitigated HSD-induced disruptions in gut metabolite composition and abundance. Among the altered metabolic features, bile acid-related metabolites showed particularly prominent changes and may be associated with the antihypertensive effects of PE under the present experimental conditions.

### 3.7 Associations among gut microbiota, metabolites, FXR/CYP8B1, and BP-Related factors

**FIGURE 7:**
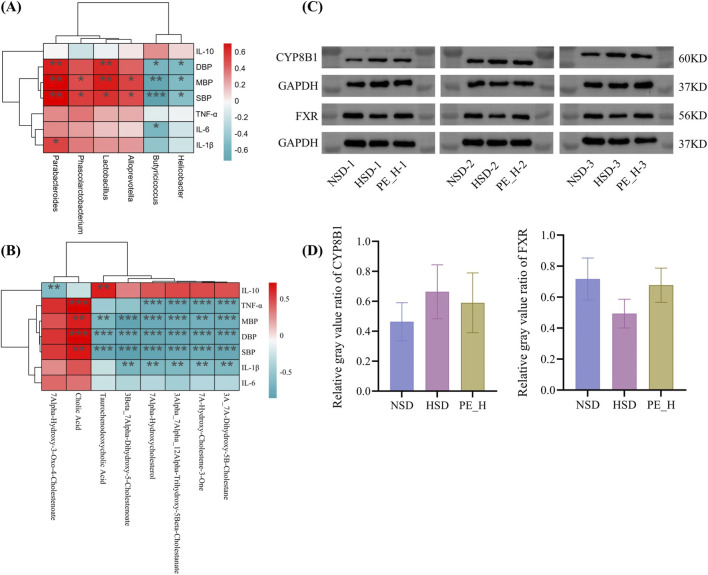
Associations of Gut Microbiota, Gut Metabolites, and Hepatic FXR/CYP8B1 Expression with BP-Related Factors. **(A)** The correlations between bacterial genera and BP metrics; **(B)** The correlations between gut metabolites and BP-related factors; **(C)** Representative western blot bands of hepatic FXR and CYP8B1 in the NSD, HSD, and PE_H groups; **(D)** Quantitative analysis of hepatic FXR and CYP8B1 protein expression normalized to the internal control.

The previous analyses suggested that both gut microbiota alterations and gut metabolic changes were associated with the protective effects of PE under HSD-induced hypertensive conditions. To further clarify these associations, we performed two complementary Spearman correlation analyses. First, Figure 7A examined the relationships between differential bacterial genera and BP-related as well as inflammatory parameters, in order to evaluate the potential links between gut microbiota composition and host phenotypes. Second, Figure 7B focused on the relationships between differential gut metabolites, particularly bile acid-related metabolites, and BP-related as well as inflammatory parameters, in order to assess the potential contribution of metabolic alterations to the observed phenotypic changes.

At the microbiota level, correlation analysis between bacterial genera and BP-related factors revealed several significant associations (Figure 7A). BP levels were positively correlated with the relative abundance of Butyricicoccus (P < 0.01) and negatively correlated with the abundance of *Lactobacillus* and Parabacteroides (P < 0.01). Furthermore, inflammatory markers exhibited strong associations: IL-6 levels were negatively correlated with Butyricicoccus abundance, whereas IL-1β levels were positively correlated with Parabacteroides abundance. These findings underscore the intricate microbiota-inflammation-BP axis potentially modulated by HSD and PE extract. At the metabolite level, correlation analysis between differential gut metabolites and BP-related factors also revealed significant associations, particularly for bile acid-related metabolites (Figure 7B). BP levels (SBP/DBP/MBP) were strongly positively correlated with the abundance of cholic acid (P < 0.001) and inversely correlated with several bile acid metabolites, including 7α-Hydroxycholesterol, 3α,7α-Dihydroxy-5β-Cholestane, 7α-Hydroxy-3-Oxo-4-Cholestenoate, 3α,7α,12α-Trihydroxy-5β-Cholestanate, and 3β,7α-Dihydroxy-5-Cholestenoate (P < 0.001). Elevated levels of TNF-α and IL-1β were also significantly associated with variations in these bile acid metabolites.

Based on the above correlation analysis, we noted that bile acid-related metabolites, particularly cholic acid, were closely associated with BP-related parameters, suggesting that bile acid dysregulation may be involved in HSD-induced BP abnormalities. Given that FXR is a key bile acid-sensing receptor involved in bile acid homeostasis, and CYP8B1 is closely related to bile acid composition, particularly cholic acid-associated metabolic changes, we further examined the hepatic protein expression of FXR and CYP8B1 to provide additional host-side evidence linking PE intervention, bile acid metabolic alterations, and BP-related improvement. Compared with the NSD group, the HSD group showed markedly decreased FXR expression and increased CYP8B1 expression, suggesting disruption of bile acid-related host regulatory signaling. PE_H intervention partially reversed these changes, as evidenced by restoration of FXR expression and reduction of CYP8B1 expression (Figure 7C). Quantitative analysis showed that normalized FXR expression decreased from 1.00 in the NSD group to 0.687 ± 0.047 in the HSD group, and was restored to 0.947 ± 0.018 after PE_H treatment. In contrast, normalized CYP8B1 expression increased from 1.00 in the NSD group to 1.439 ± 0.032 in the HSD group, and was reduced to 1.264 ± 0.088 in the PE_H group (Figure 7D). These results provide additional host-side support for the involvement of bile acid-related regulatory signaling in the protective effects of PE under HSD-induced hypertensive conditions.

Taken together, these findings indicate that gut microbiota alterations, bile acid-related metabolic changes, and hepatic FXR/CYP8B1 expression were all associated with BP-related phenotypes under the present experimental conditions.

## 4 Discussion

HSD, a well-established environmental risk factor, contributes significantly to the onset and progression of hypertension by not only increasing BP through sodium and water retention but also disrupting cardiovascular, renal, and immune system function through complex biological pathways (Luo et al., 2024). In this study, we observed that HSD markedly elevated the BP of salt-sensitive rats, exacerbated inflammatory responses, and induced pathological damage in vascular and renal tissues. Notably, the use of the salt-sensitive rat model in this study offers irreplaceable advantages and research value: compared to conventional rodent models, its core strength lies in the high degree of alignment between its pathological characteristics and human salt-sensitive hypertension. Specifically, this model authentically recapitulates the distinct BP elevation pattern observed in salt-sensitive humans following high-salt intake, as well as the accompanying target organ damage processes such as vascular endothelial injury and renal filtration dysfunction (Jiang et al., 2020). This unique capability provides a more clinically relevant animal experimental basis for translating our findings into real-world applications.

PE extract, a natural product, has gained significant attention for its potential to mitigate these effects (Chen W. et al., 2024). Our findings revealed that PE extract produced a pronounced antihypertensive effect in HSD-induced hypertension, showing an initial dose-dependent trend. Multiple studies suggest that plant extracts exert antihypertensive effects through various mechanisms, including anti-inflammatory, antioxidant, and vasodilatory pathways (Boy et al., 2021; Calvo et al., 2022; Cao et al., 2021). The antihypertensive action of PE extract appears to involve the suppression of inflammatory factor release. Previous research has identified the nuclear factor-κB pathway as a central mediator of the anti-inflammatory effects of plant extracts, achieved through macrophage inhibition and reduced cytokine overproduction (Liu et al., 2022). By targeting these pathways, PE extract likely attenuates inflammation in HSD-induced hypertension. However, the inflammatory panel assessed in the present study was limited to several representative cytokines and does not fully capture the broader inflammatory network involved in vascular remodeling. Furthermore, histological analyses using HE staining revealed significant restoration of vascular and renal tissue integrity in PE-treated rats, as evidenced by reductions in fibrosis and cellular damage. These findings suggest that PE extract exerts protective effects by enhancing vascular endothelial function, minimizing kidney injury, and inhibiting fibrotic processes in hypertensive conditions.

Emerging research highlights gut health as a critical determinant in the development of chronic diseases, including hypertension. Impairment of intestinal barrier function has been linked to HSD-induced hypertension, with disruptions in the barrier allowing endotoxins and pro-inflammatory factors to enter the bloodstream, thereby triggering systemic inflammation and elevating BP (Jin et al., 2022). In this study, PE extract significantly alleviated HSD-induced intestinal damage. Histological analysis demonstrated that the structural integrity of intestinal villi was restored, and preservation of the intestinal barrier was evident in the PE intervention group. Immunofluorescence and qPCR analyses further supported these findings, showing restored expression levels of the tight junction protein ZO-1 and reduced overexpression of Claudin-2. Together, these results suggest that PE extract may be associated with partial improvement of intestinal barrier-related alterations under HSD conditions. However, because functional permeability assays and a more comprehensive tight junction protein panel were not included, the protective effect of PE on intestinal barrier function still requires further validation.

Gut microbiota, a key player in host immunity and metabolic homeostasis, has also been implicated in the pathogenesis of hypertension. Dysbiosis of the gut microbiota, characterized by shifts in bacterial diversity and composition, has been shown to disrupt immune function and metabolic balance (Zhao et al., 2023; Mell et al., 2015; Wilck et al., 2017). Our 16S rDNA high-throughput sequencing results revealed that HSD significantly altered the diversity and structure of gut microbiota in salt-sensitive rats, notably decreasing the ratio of Bacteroidetes to Firmicutes. Intervention with high doses of PE extract restored microbial balance, significantly improving gut microbiota diversity and community structure. These findings suggest that PE extract may alleviate HSD-induced hypertension by regulating gut microbiota composition and restoring gut microecological balance.

In addition to influencing gut microbiota, microbial metabolites are known to play a pivotal role in systemic health. Among these, bile acid metabolites have emerged as critical regulators of hypertension pathophysiology (Ishimwe et al., 2022). More specifically, the present metabolomics data suggested that HSD was associated with an increase in cholic acid and a decrease in several other bile acid-related metabolites, whereas PE intervention partially reversed these changes. Notably, cholic acid showed a significant positive correlation with BP, while several bile acid-related intermediates or derivatives were inversely correlated with BP. These findings suggest that PE may be associated with partial restoration of bile acid compositional balance under HSD conditions. The additional hepatic FXR and CYP8B1 results further support the involvement of host bile acid regulatory signaling. However, other HSD-relevant metabolic pathways, including short-chain fatty acids and TMAO-related metabolism (Bier et al., 2018; Vacca et al., 2026; Wang S. et al., 2025), were not specifically examined in the present study and warrant further investigation.

**FIGURE 8:**
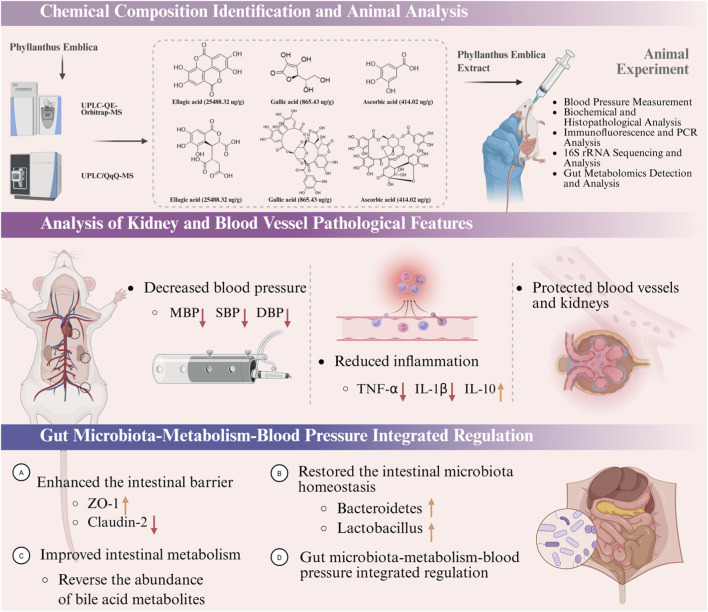
The operation and related conclusions of this experiment.

In summary, while some of the present findings confirm previous observations linking HSD to BP elevation, inflammation, gut microbiota dysbiosis, and metabolic disturbances, the more novel aspects of this study include the integrated evaluation of PE in a salt-sensitive hypertension model, the identification of bile acid-related metabolic alterations associated with PE intervention, and the additional host-side support provided by hepatic FXR/CYP8B1 expression changes. Several limitations should nevertheless be noted. The observed associations among gut microbiota, bile acid-related metabolites, and BP-related parameters remain correlative, as no FMT, germ-free, probiotic, FXR/TGR5 intervention, or LPS-related validation was performed. In addition, only two PE doses were tested, without pharmacokinetic analysis, detailed dose–response evaluation, or a standard antihypertensive positive control. Finally, the use of only male rats, the modest sample size, and the single 8-week end-point design may limit generalizability and prevent clarification of the temporal sequence of the observed changes. Overall, future studies incorporating larger sample sizes, additional dose gradients, longitudinal sampling, and more advanced experimental approaches for functional validation will be needed to further clarify the causal mechanisms and translational potential of PE in HSD-induced hypertension. The operation and related conclusions of this experiment are summarized in Figure 8.

## 5 Conclusion

This study used a salt-sensitive rat model to evaluate the protective effects of PE extract against HSD-induced hypertension and to explore its potential underlying mechanisms. The results showed that PE extract attenuated HSD-induced hypertensive phenotypes, as reflected by reduced BP, ameliorated inflammatory responses, partial improvement of renal and vascular injury, and alleviation of intestinal barrier-related alterations, with more pronounced effects observed in the high-dose group. Further analyses of gut microbiota and metabolites suggested that PE intervention was associated with partial remodeling of HSD-induced gut microbiota dysbiosis and prominent changes in gut metabolic profiles, particularly in bile acid-related metabolites. In addition, the altered hepatic expression of FXR and CYP8B1 provided further host-side support for the involvement of bile acid-related regulatory signaling in the protective effects of PE. Taken together, these findings provide preliminary preclinical evidence that PE may exert protective effects in HSD-induced hypertension, potentially in association with modulation of the gut microbiota–metabolite–host axis, and warrant further mechanistic and translational investigation.

## Data Availability

The original contributions presented in the study are included in the article/Supplementary Material, further inquiries can be directed to the corresponding authors.
