# Supplementary material for: Potential protective effects of Phyllanthus emblica L. extract on high-salt diet-induced hypertension: a combined analysis of gut microbiota and metabolomics
Source: Front Pharmacol. 2026 Jul 7;17:1728643. doi: 10.3389/fphar.2026.1728643 (PMC13385120; doi:10.3389/fphar.2026.1728643)
Supplement: Supplementary file 1 [file Table1.docx]

Table S1: Identified Chemical Constituents in PE Extract

| **Metabolite** | **HMDB SuperClass** | **HMDB Class** | **HMDB SubClass** |
| --- | --- | --- | --- |
| Rutin | Phenylpropanoids and polyketides | Flavonoids | Flavonoid glycosides |
| Quercitrin | Phenylpropanoids and polyketides | Flavonoids | Flavonoid glycosides |
| Astilbin | Phenylpropanoids and polyketides | Flavonoids | Flavonoid glycosides |
| Myricitrin | Phenylpropanoids and polyketides | Flavonoids | Flavonoid glycosides |
| Isoquercitrin | Phenylpropanoids and polyketides | Flavonoids | Flavonoid glycosides |
| Astragalin | Phenylpropanoids and polyketides | Flavonoids | Flavonoid glycosides |
| Petunidin 3-galactoside | Phenylpropanoids and polyketides | Flavonoids | Flavonoid glycosides |
| Quercetin 7-rhamnoside | Phenylpropanoids and polyketides | Flavonoids | Flavonoid glycosides |
| Isorhamnetin 3-galactoside | Phenylpropanoids and polyketides | Flavonoids | Flavonoid glycosides |
| Quercetin 3-O-neohesperidoside | Phenylpropanoids and polyketides | Flavonoids | Flavonoid glycosides |
| Naringenin | Phenylpropanoids and polyketides | Flavonoids | Flavans |
| Pinocembrin | Phenylpropanoids and polyketides | Flavonoids | Flavans |
| Myricetin | Phenylpropanoids and polyketides | Flavonoids | Flavones |
| Quercetin | Phenylpropanoids and polyketides | Flavonoids | Flavones |
| tricetin | Phenylpropanoids and polyketides | Flavonoids | Flavones |
| Morin | Phenylpropanoids and polyketides | Flavonoids | Flavones |
| 7,8-Dihydroxyflavone | Phenylpropanoids and polyketides | Flavonoids | Flavones |
| Delphinidin | Phenylpropanoids and polyketides | Flavonoids | Hydroxyflavonoids |
| Apigeninidin | Phenylpropanoids and polyketides | Flavonoids | Hydroxyflavonoids |
| Sakuranetin | Phenylpropanoids and polyketides | Flavonoids | O-methylated flavonoids |
| Isosakuranetin | Phenylpropanoids and polyketides | Flavonoids | O-methylated flavonoids |
| Scopoletin | Phenylpropanoids and polyketides | Coumarins and derivatives | Hydroxycoumarins |
| 4-methylumbelliferone | Phenylpropanoids and polyketides | Coumarins and derivatives | Hydroxycoumarins |
| afzelin | Phenylpropanoids and polyketides | Coumarins and derivatives | Pyranocoumarins |
| herniarin | Phenylpropanoids and polyketides | Coumarins and derivatives | _ |
| Ferulic Acid | Phenylpropanoids and polyketides | Cinnamic acids and derivatives | Hydroxycinnamic acids and derivatives |
| isoferulic acid | Phenylpropanoids and polyketides | Cinnamic acids and derivatives | Hydroxycinnamic acids and derivatives |
| Trans-Cinnamic acid | Phenylpropanoids and polyketides | Cinnamic acids and derivatives | Cinnamic acids |
| Isoliquiritigenin | Phenylpropanoids and polyketides | Linear 1,3-diarylpropanoids | Chalcones and dihydrochalcones |
| Licochalcone B | Phenylpropanoids and polyketides | Linear 1,3-diarylpropanoids | Chalcones and dihydrochalcones |
| Bavachalcone | Phenylpropanoids and polyketides | Linear 1,3-diarylpropanoids | Chalcones and dihydrochalcones |
| Ellagic acid | Phenylpropanoids and polyketides | Tannins | Hydrolyzable tannins |
| Corilagin | Phenylpropanoids and polyketides | Tannins | Hydrolyzable tannins |
| Geraniin | Phenylpropanoids and polyketides | Tannins | Hydrolyzable tannins |
| Chebulagic acid | Phenylpropanoids and polyketides | Tannins | Hydrolyzable tannins |
| Chebulic acid | Phenylpropanoids and polyketides | Tannins | Hydrolyzable tannins |
| Daidzein | Phenylpropanoids and polyketides | Isoflavonoids | Isoflav-2-enes |
| 5,7-DIHYDROXYISOFLAVONE | Phenylpropanoids and polyketides | Isoflavonoids | Isoflav-2-enes |
| Rhapontigenin | Phenylpropanoids and polyketides | Stilbenes | _ |
| Isorhapontigenin | Phenylpropanoids and polyketides | Stilbenes | _ |
| Piceatannol | Phenylpropanoids and polyketides | Stilbenes | _ |
| p-Coumaraldehyde | Phenylpropanoids and polyketides | Cinnamaldehydes | _ |
| Suberosin | Phenylpropanoids and polyketides | Coumarins and derivatives | _ |
| Caffeine | Organoheterocyclic compounds | Imidazopyrimidines | Purines and purine derivatives |
| Paraxanthine | Organoheterocyclic compounds | Imidazopyrimidines | Purines and purine derivatives |
| Theophylline | Organoheterocyclic compounds | Imidazopyrimidines | Purines and purine derivatives |
| Theobromine | Organoheterocyclic compounds | Imidazopyrimidines | Purines and purine derivatives |
| Loliolide | Organoheterocyclic compounds | Benzofurans | _ |
| Noreugenin | Organoheterocyclic compounds | Benzopyrans | 1-benzopyrans |
| Atractylodin | Organoheterocyclic compounds | Heteroaromatic compounds | _ |
| Ligustilide | Organoheterocyclic compounds | Isobenzofurans | _ |
| Cafestol | Organoheterocyclic compounds | Naphthofurans | _ |
| Riboflavin | Organoheterocyclic compounds | Pteridines and derivatives | Alloxazines and isoalloxazines |
| Maltol | Organoheterocyclic compounds | Pyrans | Pyranones and derivatives |
| Niacinamide | Organoheterocyclic compounds | Pyridines and derivatives | Pyridinecarboxylic acids and derivatives |
| Pergolide | Organoheterocyclic compounds | Quinolines and derivatives | Indoloquinolines |
| Ginsenoside Ro | Lipids and lipid-like molecules | Prenol lipids | Terpene glycosides |
| Kahweol | Lipids and lipid-like molecules | Prenol lipids | Monoterpenoids |
| Steviol | Lipids and lipid-like molecules | Prenol lipids | Diterpenoids |
| Atractylenolide II | Lipids and lipid-like molecules | Prenol lipids | Terpene lactones |
| Lupenone | Lipids and lipid-like molecules | Prenol lipids | Triterpenoids |
| Citramalic acid | Lipids and lipid-like molecules | Fatty Acyls | Fatty acids and conjugates |
| Azelaic acid | Lipids and lipid-like molecules | Fatty Acyls | Fatty acids and conjugates |
| Sebacic acid | Lipids and lipid-like molecules | Fatty Acyls | Fatty acids and conjugates |
| Ursolic acid | Lipids and lipid-like molecules | Prenol lipids | Triterpenoids |
| Mestranol | Lipids and lipid-like molecules | Steroids and steroid derivatives | Estrane steroids |
| Pipecolic acid | Organic acids and derivatives | Carboxylic acids and derivatives | Amino acids, peptides, and analogues |
| Citric acid | Organic acids and derivatives | Carboxylic acids and derivatives | Tricarboxylic acids and derivatives |
| Gamma-Aminobutyric acid | Organic acids and derivatives | Carboxylic acids and derivatives | Amino acids, peptides, and analogues |
| L-Proline | Organic acids and derivatives | Carboxylic acids and derivatives | Amino acids, peptides, and analogues |
| Isocitric acid | Organic acids and derivatives | Carboxylic acids and derivatives | Tricarboxylic acids and derivatives |
| Methylmalonic acid | Organic acids and derivatives | Carboxylic acids and derivatives | Dicarboxylic acids and derivatives |
| Succinic acid | Organic acids and derivatives | Carboxylic acids and derivatives | Dicarboxylic acids and derivatives |
| Pyroglutamic acid | Organic acids and derivatives | Carboxylic acids and derivatives | Amino acids, peptides, and analogues |
| Malic acid | Organic acids and derivatives | Hydroxy acids and derivatives | Beta hydroxy acids and derivatives |
| 3-Hydroxyglutaric acid | Organic acids and derivatives | Hydroxy acids and derivatives | Beta hydroxy acids and derivatives |
| D-Galactose | Organic oxygen compounds | Organooxygen compounds | Carbohydrates and carbohydrate conjugates |
| D-fructose | Organic oxygen compounds | Organooxygen compounds | Carbohydrates and carbohydrate conjugates |
| D-Tagatose | Organic oxygen compounds | Organooxygen compounds | Carbohydrates and carbohydrate conjugates |
| Panthenol | Organic oxygen compounds | Organooxygen compounds | Alcohols and polyols |
| Glucobrassicin | Organic oxygen compounds | Organooxygen compounds | Carbohydrates and carbohydrate conjugates |
| Kobusone | Organic oxygen compounds | Organooxygen compounds | Carbonyl compounds |
| Embelin | Organic oxygen compounds | Organooxygen compounds | Carbonyl compounds |
| 4-Hydroxyglucobrassicin | Organic oxygen compounds | Organooxygen compounds | Carbohydrates and carbohydrate conjugates |
| 4-Methoxyglucobrassicin | Organic oxygen compounds | Organooxygen compounds | Carbohydrates and carbohydrate conjugates |
| Glucose | Organic oxygen compounds | Organooxygen compounds | Carbohydrates and carbohydrate conjugates |
| Ascorbic acid | Organic oxygen compounds | Organooxygen compounds | Carbohydrates and carbohydrate conjugates |
| Hippuric acid | Benzenoids | Benzene and substituted derivatives | Benzoic acids and derivatives |
| Benzoic acid | Benzenoids | Benzene and substituted derivatives | Benzoic acids and derivatives |
| Gallic acid | Benzenoids | Benzene and substituted derivatives | Benzoic acids and derivatives |
| Methyl gallate | Benzenoids | Benzene and substituted derivatives | Benzoic acids and derivatives |
| Rubiadin | Benzenoids | Anthracenes | Anthraquinones |
| 3,4-Dihydroxymandelic acid | Benzenoids | Phenols | Benzenediols |
| Trigonelline | Alkaloids and derivatives | _ | _ |
| Ajmaline | Alkaloids and derivatives | Ajmaline-sarpagine alkaloids | _ |
| Phytosphingosine | Organic nitrogen compounds | Organonitrogen compounds | Amines |
