# Supplementary material for: Potential protective effects of Phyllanthus emblica L. extract on high-salt diet-induced hypertension: a combined analysis of gut microbiota and metabolomics
Source: Front Pharmacol. 2026 Jul 7;17:1728643. doi: 10.3389/fphar.2026.1728643 (PMC13385120; doi:10.3389/fphar.2026.1728643)
Supplement: Supplementary file 2 [file Table2.docx]

| Table S2. The information of 16S rDNA reads |
| --- |

| Sample ID | Raw Reads | Clean Reads | Effective Reads | GC (%) | Q20 (%) | Q30 (%) | Effective (%) |
| --- | --- | --- | --- | --- | --- | --- | --- |
| NC-1 | 79720 | 79478 | 75751 | 53.97 | 98.99 | 95.99 | 95.02 |
| NC-2 | 79710 | 79457 | 74996 | 53.91 | 99.04 | 96.09 | 94.09 |
| NC-3 | 80138 | 79864 | 75914 | 54 | 99.01 | 96.02 | 94.73 |
| NC-4 | 79886 | 79656 | 74729 | 54.51 | 99 | 96.01 | 93.54 |
| NC-5 | 80166 | 79939 | 76156 | 53.69 | 99.03 | 96.12 | 95 |
| NC-6 | 80139 | 79876 | 74895 | 53.46 | 98.96 | 95.88 | 93.46 |
| MD-1 | 79926 | 79653 | 77533 | 54.17 | 99.05 | 96.16 | 97.01 |
| MD-2 | 80215 | 79954 | 76671 | 53.63 | 99.02 | 96.06 | 95.58 |
| MD-3 | 80042 | 79813 | 76353 | 54.28 | 99.04 | 96.15 | 95.39 |
| MD-4 | 80214 | 79977 | 74515 | 53.8 | 99.06 | 96.2 | 92.9 |
| MD-5 | 80169 | 79945 | 76034 | 54.2 | 99.05 | 96.17 | 94.84 |
| MD-6 | 80058 | 79840 | 77090 | 55.18 | 98.99 | 95.97 | 96.29 |
| PE_H-1 | 79866 | 79597 | 76756 | 53.75 | 99.03 | 96.11 | 96.11 |
| PE_H-2 | 80076 | 79827 | 75819 | 53.15 | 98.92 | 95.71 | 94.68 |
| PE_H-3 | 79864 | 79600 | 77124 | 53.03 | 98.92 | 95.76 | 96.57 |
| PE_H-4 | 80213 | 79895 | 77190 | 52.91 | 98.92 | 95.74 | 96.23 |
| PE_H-5 | 79744 | 79524 | 76152 | 54.42 | 98.94 | 95.79 | 95.5 |
| PE_H-6 | 79461 | 79275 | 76506 | 52.93 | 99.03 | 96.08 | 96.28 |
