# Supplementary material for: Potential protective effects of Phyllanthus emblica L. extract on high-salt diet-induced hypertension: a combined analysis of gut microbiota and metabolomics
Source: Front Pharmacol. 2026 Jul 7;17:1728643. doi: 10.3389/fphar.2026.1728643 (PMC13385120; doi:10.3389/fphar.2026.1728643)
Supplement: Supplementary file 3 [file Table5.docx]

| Table S5. Differential metabolites in gut | | | | | |
| --- | --- | --- | --- | --- | --- |
| NSD vs. HSD | FC | P | HSD vs. PE_H | FC | P |
| 4-Hydroxyphenyllactic Acid | 0.60982 | 5.43E-11 | Nordihydroguaiaretic Acid | 5.8625 | 2.60E-12 |
| Acetylleucine | 2.797 | 7.59E-11 | Atractylenolide I | 5.6543 | 2.97E-12 |
| Carnosine | 2.253 | 1.55E-08 | 16Alpha-Hydroxyestrone | 5.7202 | 5.19E-11 |
| Alpha-Dimorphecolic Acid | 0.18942 | 2.02E-08 | Gibberellic Acid | 6.5095 | 6.15E-11 |
| Ricinoleic Acid | 0.10969 | 2.18E-08 | Americine | 994.59 | 7.41E-11 |
| 17Z-Hexacosenoic Acid | 0.057378 | 2.53E-08 | (R)-Oxypeucedanin | 1503 | 7.96E-09 |
| 4-Pyridoxic Acid | 0.35541 | 3.31E-08 | 16-Oxoestrone | 1095.8 | 8.32E-09 |
| Secalciferol | 0.12432 | 4.32E-08 | 4-Hydroxyphenyllactic Acid | 1.6586 | 1.75E-08 |
| Butyrylcarnitine | 0.5385 | 4.36E-08 | 5-Hexyl-2-Furanoctanoic Acid | 18.84 | 2.00E-08 |
| Demissidine | 0.17763 | 6.31E-08 | Gibberellin A5 | 87.81 | 2.28E-08 |
| 5Beta-Cholanic Acid | 0.1019 | 1.06E-07 | 3-Methyl-1-Butylamine | 0.46353 | 4.81E-08 |
| Glutaconic Acid | 1.757 | 1.22E-07 | 3-Methyl-2-Oxovaleric Acid | 1.8458 | 6.42E-08 |
| 3-Dehydro-6-Deoxoteasterone | 0.14576 | 1.58E-07 | Zearalenone | 241.63 | 7.93E-08 |
| Soyasapogenol D | 0.19134 | 2.15E-07 | 4-Amino-3-Hydroxybutyric Acid | 2.0869 | 1.65E-07 |
| Mogrol | 0.15718 | 2.18E-07 | Isokobusone | 1.2474 | 1.80E-07 |
| Penitrem D | 0.60434 | 2.27E-07 | (N-Acetylneuraminosyl (A2-6) Lactosamine) | 0.30584 | 2.60E-07 |
| 9,10-Dhome | 1.3451 | 2.82E-07 | Mammeisin | 2.893 | 3.01E-07 |
| Isokobusone | 0.41043 | 3.33E-07 | L-Methionine | 1.6096 | 3.63E-07 |
| Stercobilin | 0.017136 | 3.78E-07 | 4-Pyridoxic Acid | 2.1824 | 5.68E-07 |
| 13,16,19-Docosatrienoic Acid | 0.1932 | 3.81E-07 | Sclareol | 159.44 | 8.63E-07 |
| Argininosuccinic Acid | 2.5918 | 3.92E-07 | Benzoic Aldehyde | 1.4941 | 1.29E-06 |
| 7Alpha-Hydroxycholesterol | 0.16505 | 4.73E-07 | L-Phenylalanine | 1.527 | 1.38E-06 |
| Artemisin | 9.5098 | 5.07E-07 | 1-Palmitoyl-Sn-Glycero-3-Phosphocholine | 0.76054 | 2.17E-06 |
| Kynurenine | 0.31857 | 5.75E-07 | Benzyl Alcohol | 0.44957 | 2.42E-06 |
| 6-Ethylchenodeoxycholic Acid | 0.18041 | 5.92E-07 | Ectoine | 0.25539 | 2.66E-06 |
| 3A,7A-Dihydroxy-5B-Cholestane | 0.17477 | 6.29E-07 | Demissidine | 2.1815 | 2.91E-06 |
| 9-Tetradecenoic Acid | 0.14167 | 8.37E-07 | Salicylaldehyde | 1.6879 | 3.17E-06 |
| Palmitoleic Acid | 0.061876 | 8.95E-07 | Candoxatrilat | 3.4494 | 4.10E-06 |
| 6-Deoxoteasterone | 0.15504 | 9.54E-07 | Cinnamic Acid | 1.5684 | 4.15E-06 |
| Protopanaxatriol | 0.19528 | 1.19E-06 | Phenol | 1.4994 | 4.22E-06 |
| Epothilone C | 1.6226 | 1.28E-06 | Vinblastine | 18.086 | 4.76E-06 |
| Quinaldic Acid | 0.38557 | 1.44E-06 | Azelaic Acid | 0.43625 | 6.90E-06 |
| Amikacin | 1.8894 | 1.80E-06 | 2-Hydroxycinnamic Acid | 1.6515 | 6.94E-06 |
| 1-[(4-Amino-3-Methylphenyl) Methyl]-5-(2,2-Diphenylacetyl)-6,7-Dihydro-4H-Imidazo[4,5-C] Pyridine-6-Carboxylic Acid | 1.8442 | 1.81E-06 | Resolvin D1 | 0.63021 | 8.28E-06 |
| Sclareol | 0.055463 | 1.91E-06 | L-Histidine | 1.3306 | 8.44E-06 |
| 24-Epi-Brassinolide | 0.14731 | 2.36E-06 | Octopamine | 1.5972 | 8.61E-06 |
| Linoleic Acid | 0.27886 | 2.46E-06 | Choline Phosphate | 0.64011 | 9.21E-06 |
| Serotonin | 0.76979 | 3.41E-06 | Levoglucosan | 1.4604 | 1.02E-05 |
| Phenyl Hydrogen Sulfate | 0.58973 | 6.60E-06 | Cuminaldehyde | 0.52929 | 1.30E-05 |
| Piperidine | 1.2279 | 8.31E-06 | Epinephrine | 1.3565 | 1.36E-05 |
| Docosapentaenoic Acid | 2.7408 | 1.07E-05 | Zeranol | 2.514 | 1.78E-05 |
| 4,6-Dihydroxy-2-Quinolinecarboxylic Acid | 2.33 | 1.15E-05 | Butyrylcarnitine | 1.8181 | 1.80E-05 |
| 3Alpha,7Alpha,12Alpha-Trihydroxy-5Beta-Cholestanate | 0.13849 | 2.16E-05 | 2-Amino-3-Methylsuccinic Acid | 1.4614 | 1.81E-05 |
| Aniline | 0.49246 | 2.41E-05 | Ketoleucine | 1.9615 | 1.86E-05 |
| 3-Methyl-2-Oxovaleric Acid | 0.66234 | 2.55E-05 | 17Z-Hexacosenoic Acid | 3.9912 | 1.95E-05 |
| 5-Hydroxyindole-3-Acetic Acid | 0.17009 | 2.55E-05 | Cinncassiol C2 | 3.0117 | 2.06E-05 |
| L-Leucine | 1.2164 | 2.55E-05 | Ryanodine | 26.544 | 2.16E-05 |
| 3Beta,7Alpha-Dihydroxy-5-Cholestenoate | 0.2523 | 2.95E-05 | Proteinase Inhibitor E 64 | 1.3059 | 2.18E-05 |
| 7A-Hydroxy-Cholestene-3-One | 0.17729 | 3.54E-05 | Protopanaxatriol | 2.3026 | 2.22E-05 |
| 2-Amino-3-Methylsuccinic Acid | 0.63822 | 3.70E-05 | 3-Iodo-L-Tyrosine | 0.56612 | 2.31E-05 |
| Lpc(18:3) | 1.2735 | 3.82E-05 | Menthofuran | 0.54547 | 2.33E-05 |
| Ectoine | 9.9218 | 4.08E-05 | Natamycin | 1.9586 | 2.39E-05 |
| (N-Acetylneuraminosyl(A2-6) Lactosamine) | 3.8088 | 4.21E-05 | Phenyl Hydrogen Sulfate | 1.4112 | 2.54E-05 |
| Americine | 0.002187 | 4.33E-05 | 3''-Deamino-3''-Oxonicotianamine | 1.9332 | 2.61E-05 |
| 2'-Deoxycytidine | 0.76078 | 5.17E-05 | Xanthosine | 4.9058 | 2.63E-05 |
| Telmisartan | 1.5974 | 5.64E-05 | 13,16,19-Docosatrienoic Acid | 2.1208 | 2.77E-05 |
| 13,16-Docosadienoic Acid | 0.24368 | 6.04E-05 | 3A,7A-Dihydroxy-5B-Cholestane | 2.3713 | 3.32E-05 |
| Riboflavin | 0.72556 | 6.30E-05 | Deguelin | 0.44596 | 3.52E-05 |
| Propionylcarnitine | 0.5863 | 6.95E-05 | Ricinoleic Acid | 2.5583 | 4.11E-05 |
| Methaqualone | 0.66929 | 7.69E-05 | Propionylcarnitine | 1.7707 | 4.56E-05 |
| Barringtogenol C | 0.17822 | 7.93E-05 | Ethyl 3-Aminobenzoate | 1.2863 | 4.65E-05 |
| Resolvin D1 | 1.6582 | 8.96E-05 | Linoleic Acid | 1.6673 | 4.97E-05 |
| Beta-Tocopherol | 0.18042 | 9.07E-05 | L-Threonine | 1.4956 | 5.26E-05 |
| 5-Hexyl-2-Furanoctanoic Acid | 0.36767 | 0.000113 | Chlorobenzene | 0.73166 | 5.56E-05 |
| Ginsenoside C-K | 0.28549 | 0.000117 | Stercobilin | 18.46 | 5.64E-05 |
| 9-Hpode | 0.36559 | 0.000135 | Pantothenic Acid | 1.3895 | 5.92E-05 |
| Gibberellin A5 | 0.20742 | 0.000137 | Kukoamine B | 1.3334 | 7.23E-05 |
| Choline | 1.3139 | 0.000177 | Eucalyptol | 0.80918 | 7.50E-05 |
| Xanthosine | 0.24526 | 0.000181 | 7Alpha-Hydroxycholesterol | 2.2532 | 7.83E-05 |
| Carisoprodol | 4.4298 | 0.000189 | Phenaceturic Acid | 1.7193 | 8.04E-05 |
| Carboprost | 1.8788 | 0.000214 | Gpcho(18:3/22:4) | 0.67106 | 8.45E-05 |
| Palmitoylcarnitine | 1.4037 | 0.000215 | 6-Deoxoteasterone | 2.586 | 9.00E-05 |
| Isorhapontin | 0.17503 | 0.000216 | Zearalenol | 213.91 | 9.13E-05 |
| Bullatanocin | 0.23913 | 0.000236 | Penitrem D | 1.2992 | 9.16E-05 |
| L-Methionine | 0.82239 | 0.000246 | Zearalanone | 2.7815 | 9.50E-05 |
| Hyocholic Acid | 8.0807 | 0.000333 | Cytosine | 1.4249 | 9.99E-05 |
| 2-Hydroxy-6-Pentadecylbenzoic Acid | 0.32721 | 0.000345 | Phaseic Acid | 2.3732 | 9.99E-05 |
| Buprenorphine | 0.62573 | 0.000476 | Dodecanoic Acid | 0.68869 | 0.000103 |
| 3-Methyl-1-Butylamine | 1.8051 | 0.000503 | 6-Ethylchenodeoxycholic Acid | 2.1147 | 0.000108 |
| N-Acetyl-D-Glucosamine | 0.65719 | 0.000534 | 9-Tetradecenoic Acid | 2.6682 | 0.000116 |
| N4-Acetylcytidine | 3.0093 | 0.000609 | 3-Dehydro-6-Deoxoteasterone | 2.1642 | 0.000123 |
| Pe (36:4) | 1.4516 | 0.000621 | 2-(Methylthio)Benzothiazole | 0.71135 | 0.000124 |
| 1,4-Dihydro-1-Methyl-4-Oxo-3-Pyridinecarboxamide | 0.6889 | 0.000627 | Beta-Propiolactone | 1.3956 | 0.000131 |
| Candoxatrilat | 0.43842 | 0.000639 | Palmitoleic Acid | 3.602 | 0.000136 |
| Urobilin | 1.546 | 0.000642 | L-Isoleucine | 1.4556 | 0.000138 |
| Cholic Acid | 2.9914 | 0.00065 | Virginiamycin M1 | 1.594 | 0.00014 |
| Mammeisin | 0.43051 | 0.00066 | Soyasapogenol D | 1.8798 | 0.000148 |
| Imperatorin | 1.8361 | 0.000665 | Solutol Hs 15 | 3.1062 | 0.000151 |
| Vindoline | 1.2712 | 0.000695 | D-(+)-Cellobiose | 0.56944 | 0.000155 |
| Proteinase Inhibitor E 64 | 0.70476 | 0.000722 | 2'-Deoxycytidine | 1.3361 | 0.000156 |
| Isocitric Acid | 0.70489 | 0.000881 | Abacavir | 49.603 | 0.000157 |
| Ketoleucine | 0.65596 | 0.000928 | Quinaldic Acid | 2.0676 | 0.000165 |
| Abacavir | 0.11966 | 0.000969 | Alpha-Methyl-M-Tyrosine | 0.62196 | 0.000178 |
| Endomorphin-1 | 1.6339 | 0.001097 | Pc(34:0) | 0.6986 | 0.000185 |
| Chaps | 0.52306 | 0.001258 | Amikacin | 0.69402 | 0.000201 |
| N2-Acetyl-L-Ornithine | 1.9847 | 0.001348 | Tulathromycin A | 0.52809 | 0.000205 |
| Vinblastine | 0.081 | 0.001363 | 7A-Hydroxy-Cholestene-3-One | 3.2966 | 0.000215 |
| Pc (22:5/0:0) | 1.3751 | 0.00151 | Methyltestosterone | 28.102 | 0.00023 |
| Janthitrem C | 1.3049 | 0.00156 | Epothilone C | 0.78393 | 0.000244 |
| Ryanodine | 0.068173 | 0.001612 | Pc(34:2) | 0.54361 | 0.000257 |
| Kukoamine A | 0.55717 | 0.001671 | Gitoxin | 2.3975 | 0.000278 |
| 3''-Deamino-3''-Oxonicotianamine | 0.44858 | 0.00181 | Secalciferol | 1.898 | 0.000282 |
| N-[(3A,5B,7A)-3-Hydroxy-24-Oxo-7-(Sulfooxy) Cholan-24-Yl]-Glycine | 0.60821 | 0.001815 | 4-Coumaric Acid | 1.3287 | 0.000289 |
| Tamibarotene | 0.35989 | 0.001826 | Sulfasalazine | 2.9379 | 0.000314 |
| Tyramine | 1.6027 | 0.001848 | Linalool | 0.76577 | 0.000368 |
| Pc(32:0) | 1.5444 | 0.002066 | Kukoamine A | 2.201 | 0.000383 |
| Malonic Semialdehyde | 1.4714 | 0.002094 | Latrunculin A | 1.3238 | 0.000388 |
| Methyltestosterone | 0.046964 | 0.002253 | Serotonin | 1.5574 | 0.000418 |
| Fucose | 2.512 | 0.002406 | L-Valine | 0.7036 | 0.000433 |
| Alpha-Allocryptopine | 2.0248 | 0.002587 | Cyclopamine | 0.74125 | 0.000435 |
| 3-Methylcrotonylglycine | 1.4642 | 0.002588 | Pe(36:4) | 0.69434 | 0.00045 |
| Trinexapac-Ethyl | 1.3028 | 0.002753 | Coriolic Acid | 1.9506 | 0.000456 |
| 7-Aminomethyl-7-Carbaguanine | 0.61934 | 0.002932 | Isocitric Acid | 1.4138 | 0.000458 |
| Dodecanoic Acid | 1.5729 | 0.003538 | 5'-Deoxy-5-Fluorocytidine | 1.594 | 0.000468 |
| 13-Demethyl Tacrolimus | 0.63331 | 0.003551 | Adenosine | 2.2912 | 0.000494 |
| Nonactin | 0.67846 | 0.003598 | N-Acetyl-4-O-Acetylneuraminic Acid | 0.80474 | 0.000534 |
| N-Acetyl-4-O-Acetylneuraminic Acid | 1.217 | 0.004089 | Telmisartan | 0.75288 | 0.000573 |
| Salicylaldehyde | 0.80083 | 0.004238 | Gpcho(18:1/16:0) | 0.52814 | 0.000595 |
| Mucronine D | 0.48122 | 0.004471 | 2,4-Dichlorophenol | 1.7508 | 0.000618 |
| Hexadecanedioic Acid | 1.309 | 0.004629 | Gentamicin C2 | 43.097 | 0.000648 |
| Coriolic Acid | 0.5782 | 0.004634 | 5-Androstenediol | 0.66771 | 0.000702 |
| Taurochenodeoxycholic Acid | 0.33636 | 0.004732 | Astaxanthin | 1.4193 | 0.000726 |
| Cannabigerolate | 1.9981 | 0.004838 | Kynurenine | 1.591 | 0.000756 |
| Alpha-D-Glucose | 0.76022 | 0.004948 | Trinexapac-Ethyl | 0.70442 | 0.000766 |
| 1-Palmitoyl-Sn-Glycero-3-Phosphocholine | 1.2352 | 0.005077 | Gpcho(18:1/18:0) | 0.5971 | 0.000775 |
| Cephaloglycin | 0.25908 | 0.00515 | Cyclohexanone | 0.7889 | 0.000798 |
| Troxilin B3 | 0.47548 | 0.005229 | 13-Oxoode | 1.8911 | 0.000807 |
| 4Alpha-Carboxy-5Alpha-Cholesta-8,24-Dien-3Beta-Ol | 2.5953 | 0.005294 | L-Tyrosine | 1.2402 | 0.000809 |
| Melibiose | 0.69716 | 0.005339 | 13,16-Docosadienoic Acid | 2.1573 | 0.000824 |
| L-Threonine | 0.7265 | 0.005352 | Erythrose | 1.2086 | 0.00084 |
| 1-Naphthaleneacetic Acid | 1.6036 | 0.005719 | Chaps | 1.8933 | 0.000852 |
| Stearaldehyde | 0.45146 | 0.005942 | (+/-)-Tryptophan | 1.4906 | 0.000877 |
| Alpha-Hederin | 0.44817 | 0.006127 | Pc(34:1) | 0.7059 | 0.000918 |
| Bufotalin | 0.62304 | 0.00686 | 1,5-Naphthalenediamine | 1.4156 | 0.000952 |
| Mezlocillin | 0.65026 | 0.006959 | (-)-Salsoline | 1.8634 | 0.000954 |
| Fructosyl-Lysine | 0.6241 | 0.007048 | Tamibarotene | 2.2297 | 0.0011 |
| (S)-Equol | 0.17932 | 0.007172 | Dihydroconiferyl Alcohol | 1.9378 | 0.001102 |
| 7Alpha-Hydroxy-3-Oxo-4-Cholestenoate | 4.7516 | 0.007213 | Cauloside C | 1.612 | 0.001135 |
| 3-Guanidinopropionic Acid | 1.3525 | 0.007378 | Dolichyl B-D-Glucosyl Phosphate | 0.48453 | 0.001154 |
| (+/-)-Tryptophan | 0.70946 | 0.007525 | Didemnin A | 1.479 | 0.001182 |
| Guaiazulene | 4.1388 | 0.007797 | N-Alpha-Acetyllysine | 2.7498 | 0.001183 |
| Aclacinomycin N | 1.6782 | 0.008092 | Indole-3-Carboxaldehyde | 1.3925 | 0.001303 |
| 1-Methyl-L-Histidine | 1.3331 | 0.008209 | Citrulline | 0.83285 | 0.001304 |
| L-3-Aminodihydro-2(3H)-Furanone | 0.73566 | 0.008936 | Cephaloglycin | 5.2328 | 0.00133 |
| Soyasaponin I | 1.4594 | 0.009146 | Hebevinoside I | 1.5672 | 0.001457 |
| Gentamicin C2 | 0.39705 | 0.009734 | 1-Naphthaleneacetic Acid | 0.56894 | 0.001467 |
| Octopamine | 0.77879 | 0.009748 | Uric Acid | 1.2746 | 0.001557 |
| Choline Phosphate | 1.385 | 0.009908 | 5Beta-Cholanic Acid | 1.8851 | 0.00157 |
| Oxiglutatione | 0.48695 | 0.010433 | Anisole | 1.6117 | 0.001644 |
| 21-Hydroxypregnenolone | 0.26965 | 0.011427 | Fructosyl-Lysine | 2.1671 | 0.001685 |
| 2-Hydroxycinnamic Acid | 0.81895 | 0.011571 | Kojic Acid | 0.80602 | 0.001688 |
| Spermidine | 0.68663 | 0.012389 | L-Tryptophan | 1.3738 | 0.001692 |
| Latrunculin A | 0.81363 | 0.012483 | Indolelactic Acid | 1.6649 | 0.001731 |
| Taurodeoxycholic Acid | 0.092107 | 0.012723 | Betaine | 0.70884 | 0.001743 |
| Azelaic Acid | 1.5675 | 0.012742 | Alpha-Dimorphecolic Acid | 1.5413 | 0.001774 |
| Astaxanthin | 0.70615 | 0.013495 | Bullatanocin | 2.4086 | 0.00196 |
| 3-Dehydroteasterone | 0.56869 | 0.013952 | 1,4-Dihydro-1-Methyl-4-Oxo-3-Pyridinecarboxamide | 1.3596 | 0.002135 |
| Norethindrone Acetate | 0.79993 | 0.01469 | N-Acetylserotonin | 1.9727 | 0.002168 |
| Elemicin | 1.5412 | 0.016273 | Uridine | 1.5434 | 0.002227 |
| O-Xylene | 2.086 | 0.016935 | (+)-Cis-Abscisic Aldehyde | 2.0415 | 0.00225 |
| Creatine | 1.2411 | 0.018533 | Riboflavin | 1.4626 | 0.00227 |
| Succinic Acid | 2.5725 | 0.019366 | Levetiracetam | 1.5309 | 0.002648 |
| Metconazole | 0.68245 | 0.019667 | 7-Amino-4-Methylcoumarin | 1.8123 | 0.00274 |
| L-Anserine | 2.0546 | 0.020119 | Troxilin B3 | 2.1671 | 0.002756 |
| Phaseic Acid | 0.74947 | 0.020876 | Methaqualone | 0.47732 | 0.002832 |
| Angiotensin Iii | 0.27032 | 0.020984 | Phenethylamine Glucuronide | 1.7947 | 0.002884 |
| Sphingosine | 1.2043 | 0.021472 | Pe(36:2) | 0.75301 | 0.002891 |
| Netilmicin | 0.74969 | 0.022434 | Cyclohexanecarboxylic Acid | 1.4932 | 0.00296 |
| Bufadienolide | 4.2093 | 0.023169 | 17Alpha,21-Dihydroxypregnenolone | 2.0382 | 0.00304 |
| Docosahexaenoic Acid | 1.5233 | 0.02389 | Choline Glycerophosphate | 0.73992 | 0.003085 |
| Pantetheine 4'-Phosphate | 0.26954 | 0.024651 | 2-Mercaptobenzothiazole | 0.77254 | 0.003218 |
| Uridine | 0.72903 | 0.024715 | Dodecanedioic Acid | 1.4801 | 0.003267 |
| 12-Hete | 0.30826 | 0.026226 | Spinosin | 2.0498 | 0.003431 |
| 2-Oxindole | 0.73966 | 0.026479 | Pseudouridine | 0.79904 | 0.003828 |
| 5-Androstenediol | 1.5138 | 0.028313 | Docosapentaenoic Acid | 0.76596 | 0.003841 |
| (+)-Cis-Abscisic Aldehyde | 1.7739 | 0.029129 | Gluconic Acid | 0.74251 | 0.00397 |
| Tulathromycin A | 1.5839 | 0.031704 | 1-[(4-Amino-3-Methylphenyl)Methyl]-5-(2,2-Diphenylacetyl)-6,7-Dihydro-4H-Imidazo[4,5-C]Pyridine-6-Carboxylic Acid | 0.83283 | 0.004062 |
| 1-Methylnicotinamide | 1.6385 | 0.031874 | Carnosine | 0.45651 | 0.004082 |
| Acetylcholine | 1.2093 | 0.032968 | Syringetin | 2.2232 | 0.004207 |
| 2,4-Dichlorophenol | 0.71801 | 0.034909 | Mogrol | 1.6211 | 0.004384 |
| 1,5-Naphthalenediamine | 0.83007 | 0.035686 | L-Malic Acid | 0.54104 | 0.004468 |
| Enterolactone | 1.4507 | 0.037083 | Elemicin | 0.6145 | 0.004653 |
| L-Malic Acid | 1.5904 | 0.037433 | 3Alpha,7Alpha,12Alpha-Trihydroxy-5Beta-Cholestanate | 2.2263 | 0.004715 |
| Levetiracetam | 1.3926 | 0.038342 | Pantetheine 4'-Phosphate | 4.846 | 0.004806 |
| Taxifolin | 2.0408 | 0.039284 | N2-Acetyl-L-Ornithine | 1.4044 | 0.004922 |
| Levocarnitine | 0.75066 | 0.040722 | Nitrosobenzene | 0.83088 | 0.004941 |
| 2-Oxoglutaric Acid | 0.81637 | 0.041226 | Cannabigerolate | 0.56756 | 0.005225 |
| Cauloside C | 0.64167 | 0.041499 | Taurochenodeoxycholic Acid | 1.9694 | 0.00532 |
| Alpha-Methyl-M-Tyrosine | 1.3272 | 0.04171 | (S)-Equol | 5.7722 | 0.005432 |
| Cerebronic Acid | 0.74545 | 0.041801 | Spermidine | 1.2963 | 0.005696 |
| L-Threonic Acid | 1.4323 | 0.042102 | L-Glycine | 4.695 | 0.005788 |
| Benzyl Alcohol | 1.4363 | 0.042474 | Bufotalin | 1.4743 | 0.006276 |
| Kynurenic Acid | 0.67609 | 0.043265 | Beta-Tocopherol | 2.2166 | 0.006328 |
| Pc(34:0) | 1.2029 | 0.043958 | Rhodamine 6G | 0.81808 | 0.006953 |
| Pc(34:2) | 1.4902 | 0.043998 | (R)-2-Hydroxystearic Acid | 1.5754 | 0.007031 |
| Citrulline | 1.2207 | 0.044095 | Artemisin | 0.5615 | 0.007421 |
| Ruscogenin | 2.2714 | 0.045099 | Nonaprenyl-4-Hydroxybenzoate | 0.68747 | 0.007916 |
| Menthofuran | 1.4342 | 0.047445 | 2-Hydroxybutyric Acid | 1.2437 | 0.008189 |
| Gpcho(18:1/18:0) | 1.5177 | 0.048577 | Nicotinamide | 1.5777 | 0.008811 |
|  |  |  | Lpc(17:0) | 0.82088 | 0.008815 |
|  |  |  | Pc(32:0) | 0.28779 | 0.008903 |
|  |  |  | Dihydroorotic Acid | 1.5155 | 0.009277 |
|  |  |  | Metoclopramide | 2.3167 | 0.009393 |
|  |  |  | Fluazifop | 0.71474 | 0.009802 |
|  |  |  | 3Beta,7Alpha-Dihydroxy-5-Cholestenoate | 1.7036 | 0.00997 |
|  |  |  | Gpcho(16:0/20:5) | 0.71552 | 0.010088 |
|  |  |  | Tagatose | 1.201 | 0.010195 |
|  |  |  | Alpha-Hederin | 2.0984 | 0.010543 |
|  |  |  | 3-Hydroxybenzaldehyde | 1.2331 | 0.01095 |
|  |  |  | 5-Aminovaleric Acid | 0.4537 | 0.011077 |
|  |  |  | Metconazole | 1.8564 | 0.011102 |
|  |  |  | 5-L-Glutamyl-L-Alanine | 1.3659 | 0.012086 |
|  |  |  | 12-Hete | 2.9036 | 0.012241 |
|  |  |  | 3-Methylcrotonylglycine | 1.3765 | 0.01225 |
|  |  |  | Diethanolamine | 1.2134 | 0.013021 |
|  |  |  | Asarone | 0.78754 | 0.013051 |
|  |  |  | 7-Aminomethyl-7-Carbaguanine | 1.5565 | 0.01317 |
|  |  |  | 1-(Beta-D-Ribofuranosyl)-1,4-Dihydronicotinamide | 1.2289 | 0.013176 |
|  |  |  | Thromboxane | 2.1935 | 0.013481 |
|  |  |  | 1-O-Hexadecyl-Sn-Glycero-3-Phosphocholine | 1.2693 | 0.013959 |
|  |  |  | 6-Keto-Pgf1Alpha | 0.78494 | 0.014616 |
|  |  |  | Stearaldehyde | 2.1231 | 0.014788 |
|  |  |  | 24-Epi-Brassinolide | 1.6923 | 0.014984 |
|  |  |  | Tautomycin | 1.2248 | 0.015254 |
|  |  |  | Araloside A | 0.68726 | 0.015423 |
|  |  |  | Oleandomycin | 1.5663 | 0.015788 |
|  |  |  | Urobilin | 0.5856 | 0.018976 |
|  |  |  | Ascorbic Acid | 1.4254 | 0.018976 |
|  |  |  | Indole-3-Acetaldehyde | 1.4768 | 0.019069 |
|  |  |  | Succinic Acid | 0.38837 | 0.019348 |
|  |  |  | Buprenorphine | 1.2489 | 0.020303 |
|  |  |  | Cefpodoxime | 0.83293 | 0.021837 |
|  |  |  | N-Acetylneuraminic Acid | 0.75341 | 0.022301 |
|  |  |  | Pe(36:1) | 0.42693 | 0.022974 |
|  |  |  | 21-Hydroxypregnenolone | 2.1295 | 0.023183 |
|  |  |  | Glycochenodeoxycholic Acid | 1.9475 | 0.024057 |
|  |  |  | Oxiglutatione | 1.8478 | 0.024987 |
|  |  |  | Pyrrolnitrin | 2.2093 | 0.028195 |
|  |  |  | Hordenine | 0.72352 | 0.029807 |
|  |  |  | Pyruvic Acid | 1.3438 | 0.031986 |
|  |  |  | Sucrose | 0.82167 | 0.035107 |
|  |  |  | Oxyquinoline | 1.7541 | 0.035421 |
|  |  |  | Decanoyl-L-Carnitine | 1.5899 | 0.035715 |
|  |  |  | Bergenin | 1.5202 | 0.036006 |
|  |  |  | 2-Oxindole | 1.3678 | 0.036342 |
|  |  |  | Hippuric Acid | 1.3754 | 0.036567 |
|  |  |  | Taxifolin | 1.574 | 0.039395 |
|  |  |  | L-Ornithine | 1.3098 | 0.040377 |
|  |  |  | Mucronine D | 1.5836 | 0.042638 |
|  |  |  | Netilmicin | 1.2249 | 0.043374 |
|  |  |  | Tyramine | 1.3191 | 0.044485 |
|  |  |  | 4-Hydroxybenzaldehyde | 1.4217 | 0.044583 |
|  |  |  | Cerebronic Acid | 0.76002 | 0.046882 |
|  |  |  | Angiotensin Iii | 1.374 | 0.048261 |
