# Supplementary material for: Potential protective effects of Phyllanthus emblica L. extract on high-salt diet-induced hypertension: a combined analysis of gut microbiota and metabolomics
Source: Front Pharmacol. 2026 Jul 7;17:1728643. doi: 10.3389/fphar.2026.1728643 (PMC13385120; doi:10.3389/fphar.2026.1728643)
Supplement: Supplementary file 4 [file Table3.docx]

| Table S3. Differential gut microbiota at genus levels | | | | | |
| --- | --- | --- | --- | --- | --- |
| NSD vs. HSD | FC | P | HSD vs. PE_H | FC | P |
| Methylomonas | 9.36 | 0.00029801 | [Eubacterium]_coprostanoligenes_group | 2.9934 | 4.47E-05 |
| Bifidobacterium | 0.027848 | 0.00044311 | Lachnospiraceae_NK4A136_group | 0.29401 | 0.00016487 |
| Lactobacillus | 0.30051 | 0.00054612 | Acetatifactor | 0.11111 | 0.00040596 |
| Methylotenera | 11.33 | 0.00093617 | Ruminococcaceae_UCG-014 | 1.9695 | 0.0005528 |
| Inhella | 6.9318 | 0.0011727 | [Eubacterium]_nodatum_group | 3.1939 | 0.00058755 |
| Prevotellaceae_NK3B31_group | 7.3173 | 0.0020555 | Ruminococcaceae_NK4A214_group | 3.0645 | 0.00058797 |
| Geothrix | 6.1765 | 0.0024938 | Lactobacillus | 4.5577 | 0.0010197 |
| Mucispirillum | 0.011858 | 0.0028572 | Harryflintia | 0.15276 | 0.0012063 |
| Parabacteroides | 0.2864 | 0.0035906 | Oscillibacter | 0.2946 | 0.0014196 |
| Romboutsia | 0.21094 | 0.0039034 | Bifidobacterium | 9.7727 | 0.0017273 |
| Butyricicoccus | 4.9216 | 0.0039082 | Intestinimonas | 0.30106 | 0.0031118 |
| Escherichia-Shigella | 0.21574 | 0.0071157 | [Eubacterium]_oxidoreducens_group | 0.2585 | 0.0047234 |
| Macellibacteroides | 0.57143 | 0.0072332 | Defluviitaleaceae_UCG-011 | 2.2941 | 0.0048237 |
| Prevotellaceae_UCG-003 | 0.0043441 | 0.0087641 | Akkermansia | 5.3395 | 0.0081969 |
| Candidatus_Competibacter | 0.076735 | 0.01055 | Alloprevotella | 23.113 | 0.013473 |
| Lysobacter | 2.8464 | 0.011502 | Christensenellaceae_R-7_group | 2.8139 | 0.01375 |
| Treponema_2 | 0.00089039 | 0.012687 | Lachnospiraceae_UCG-010 | 0.31282 | 0.017096 |
| Rikenellaceae_RC9_gut_group | 0.004717 | 0.015868 | Butyricicoccus | 0.41434 | 0.017242 |
| [Ruminococcus]_torques_group | 0.1 | 0.016561 | Pygmaiobacter | 0.30625 | 0.017664 |
| Helicobacter | 3.4604 | 0.017446 | Ruminiclostridium_5 | 0.44764 | 0.019763 |
| Shuttleworthia | 0.32857 | 0.01985 | Helicobacter | 0.28465 | 0.020147 |
| Phascolarctobacterium | 0.44666 | 0.02024 | Ruminococcaceae_UCG-005 | 3.1024 | 0.024731 |
| Erysipelatoclostridium | 0.22667 | 0.020642 | Phascolarctobacterium | 2.182 | 0.029016 |
| Methylomagnum | 9.2 | 0.020807 | Ruminiclostridium_9 | 0.486 | 0.030131 |
| Negativibacillus | 0.34444 | 0.026449 | Erysipelatoclostridium | 5.5882 | 0.030339 |
| Aeromonas | 0.66529 | 0.030541 | Negativibacillus | 3.129 | 0.032548 |
| Nitrospira | 0.42414 | 0.03324 | Macellibacteroides | 1.4643 | 0.035437 |
| Anaerobiospirillum | 0.0009759 | 0.035066 | Escherichia-Shigella | 4.4588 | 0.03618 |
| Faecalibaculum | 0.0082508 | 0.03527 | Ruminiclostridium | 0.38806 | 0.036926 |
| Alloprevotella | 0.035411 | 0.037943 | Desulfobulbus | 2.2297 | 0.039813 |
| Aminivibrio | 0.48 | 0.047071 | DNF00809 | 0.40976 | 0.042107 |
|  |  |  | Parabacteroides | 2.3778 | 0.047489 |
|  |  |  | Tyzzerella_3 | 0.40071 | 0.048096 |
